# Adherence of SARS‐CoV‐2 Seroepidemiologic Studies to the ROSES‐S Reporting Guideline During the COVID‐19 Pandemic

**DOI:** 10.1111/irv.13283

**Published:** 2024-07-25

**Authors:** Brianna Cheng, Emma Loeschnik, Anabel Selemon, Reza Hosseini, Jane Yuan, Harriet Ware, Xiaomeng Ma, Christian Cao, Isabel Bergeri, Lorenzo Subissi, Hannah C. Lewis, Tyler Williamson, Paul Ronksley, Rahul K. Arora, Mairead Whelan, Niklas Bobrovitz

**Affiliations:** ^1^ Temerty Faculty of Medicine University of Toronto Toronto Ontario Canada; ^2^ Department of Epidemiology and Biostatistics, Schulich School of Medicine and Dentistry Western University London Ontario Canada; ^3^ Centre for Health Informatics, Cumming School of Medicine University of Calgary Calgary Alberta Canada; ^4^ Institute of Health Policy Management and Evaluation University of Toronto Toronto Ontario Canada; ^5^ Department of Epidemic and Pandemic Prevention and Preparedness, Health Emergencies Programme World Health Organization Geneva Switzerland; ^6^ Department of Community Health Sciences, Cumming School of Medicine University of Calgary Calgary Alberta Canada; ^7^ Institute of Biomedical Engineering University of Oxford Oxford UK; ^8^ Department of Emergency Medicine, Cumming School of Medicine University of Calgary Calgary Alberta Canada

**Keywords:** guideline adherence, research design, SARS‐CoV‐2, seroepidemiologic studies

## Abstract

**Background:**

Complete reporting of seroepidemiologic studies is critical to their utility in evidence synthesis and public health decision making. The Reporting of Seroepidemiologic studies—SARS‐CoV‐2 (ROSES‐S) guideline is a checklist that aims to improve reporting in SARS‐CoV‐2 seroepidemiologic studies. Adherence to the ROSES‐S guideline has not yet been evaluated.

**Objectives:**

This study aims to evaluate the completeness of SARS‐CoV‐2 seroepidemiologic study reporting by the ROSES‐S guideline during the COVID‐19 pandemic, determine whether guideline publication was associated with reporting completeness, and identify study characteristics associated with reporting completeness.

**Methods:**

A random sample from the SeroTracker living systematic review database was evaluated. For each reporting item in the guideline, the percentage of studies that were adherent was calculated, as well as median and interquartile range (IQR) adherence across all items and by item domain. Beta regression analyses were used to evaluate predictors of adherence to ROSES‐S.

**Results:**

One hundred and ninety‐nine studies were analyzed. Median adherence was 48.1% (IQR 40.0%–55.2%) per study, with overall adherence ranging from 8.8% to 72.7%. The laboratory methods domain had the lowest median adherence (33.3% [IQR 25.0%–41.7%]). The discussion domain had the highest median adherence (75.0% [IQR 50.0%–100.0%]). Reporting adherence to ROSES‐S before and after guideline publication did not significantly change. Publication source (*p* < 0.001), study risk of bias (*p* = 0.001), and sampling method (*p* = 0.004) were significantly associated with adherence.

**Conclusions:**

Completeness of reporting in SARS‐CoV‐2 seroepidemiologic studies was suboptimal. Publication of the ROSES‐S guideline was not associated with changes in reporting practices. Authors should improve adherence to the ROSES‐S guideline with support from stakeholders.

## 1 Introduction

Measuring the prevalence of antibodies against SARS‐CoV‐2 in population blood and serum samples (seroprevalence) provides valuable estimates of true population infection, including infections in asymptomatic individuals or those who were unable to acquire diagnostic services [1]. This makes serosurveillance a valuable tool for pandemic decision‐making. Seroepidemiologic studies must be well reported to be useful, however, as information about how seroprevalence was measured (e.g., immunoassay characteristics and validation), sampling methods, and statistical techniques affects study interpretation, comparison, and application [2].

Inconsistent reporting of study methods and results was evident during the first year of the COVID‐19 pandemic [3] despite the existence of guidelines for study designs typically used to estimate seroprevalence (e.g., the “Strengthening the Reporting of Observational Studies in Epidemiology” [STROBE] guidelines) [4]. Reporting guidelines were also available for specific domains of epidemiological research, such as observational infectious disease studies (e.g., the “Strengthening the Reporting of Molecular Epidemiology for Infectious Diseases” [STROME‐ID] statement) and influenza seroepidemiology (e.g., the CONSISE “Reporting Of Sero‐Epidemiologic Studies for Influenza” [ROSES‐I]) statement. Previous studies suggest that the use of these types of guidelines improves reporting [5, 6], yet adherence to such guidelines still appears suboptimal. For instance, estimates of adherence to STROBE's 22 reporting criteria have ranged from 51.4% to 76.5% [7, 8, 9, 10], with similar estimates of adherence (50%) to STROME‐ID [11].

In June 2021, the World Health Organization (WHO) published the “Reporting of Sero‐Epidemiologic Studies—SARS‐CoV‐2” (ROSES‐S) guideline as a content‐specific extension of the STROBE checklist. ROSES‐S is a checklist of 22 criteria for the reporting of SARS‐CoV‐2 seroepidemiologic studies, regardless of study design [12].

To better understand reporting completeness among SARS‐CoV‐2 seroepidemiologic studies and determine whether ROSES‐S has changed practice, we conducted a sub‐study within a living systematic review with the following aims: (i) quantify the completeness of reporting among SARS‐CoV‐2 seroepidemiologic studies by assessing adherence to the ROSES‐S reporting guideline; (ii) assess whether specific study characteristics were associated with adherence to the ROSES‐S guideline; and (iii) determine whether implementation of the ROSES‐S guideline was associated with changes in the reporting completeness of SARS‐CoV‐2 seroepidemiologic studies.

## 2 Methods

### 2.1 Search Strategy and Eligibility Criteria

This systematic review sub‐study was reported according to the Preferred Reporting Items for Systematic Review and Meta Analysis (PRISMA) Statement [29] (Data S1) and was embedded within SeroTracker's living global systematic review of SARS‐CoV‐2 seroepidemiologic studies (PROSPERO [CRD42020183634]) [13], which is available on a public dashboard and data platform [14]. An in‐depth description of the methods, including a full search strategy, inclusion and exclusion criteria, and screening process, has been previously published [14, 15]. Briefly, a search of electronic databases, gray literature, and news media captured published and pre‐print studies, as well as institutional reports from January 1, 2020, to December 31, 2022. Direct submissions of data were also invited at SeroTracker.com.

A seroepidemiologic study was defined as estimating the prevalence of SARS‐CoV‐2 antibodies in humans through the collection and testing of serum (or proxy, such as oral fluid) specimens from a defined sample frame over a specified period of time. Eligible study designs included cross sectional studies, repeated cross‐sectional studies, and cohort studies. Studies of all languages were included if they reported the number of participants, sampling end date and week, geographic location of sampling, and a prevalence estimate. Studies in languages other than English, French, Spanish, Romanian, Persian, and Chinese were translated via Google translate machine translation.

Studies were screened using Covidence systematic review manager [16] via a two‐stage screening process by two independent reviewers. The first stage determined inclusion based on review of study title and/or abstract. The second stage determined inclusion by review of the full text. A third team member resolved discrepancies by arbitration.

### 2.2 Study Selection

We included a random time‐stratified sample (*n* = 199) of peer‐reviewed, pre‐print studies, and gray literature (government and institutional reports) from March 2020 to December 2022 to capture studies before and after the publication of the ROSES‐S guideline (June 2021). The sample size was determined based on a precision level of ±7% with 95% confidence and a 60% estimated adherence to checklist items [11, 17]. To ensure a comparable sample size before and after the ROSES‐S guideline's publication date, we created strata of six month discretized time periods and randomly sampled 34 studies from each strata. If a given month had only one included study, then we conducted additional random sampling of studies from that month to select an additional study—bringing the total to two.

### 2.3 Data Extraction

The following data was extracted by one independent reviewer using an Airtable spreadsheet tool (2022) and verified by a second reviewer from the SeroTracker team: article publication source, study design, publication month, sampling frame, sampling method, sample size, geographic scope (local, regional, national), WHO Humanitarian Response Plan (HRP) status [18], WHO Unity protocol alignment [19], journal impact factor (IF), SARS‐CoV‐2 antibody seroprevalence estimate(s), and whether the article cited the ROSES‐S guideline or other reporting guidelines. The risk of bias (RoB) (low, moderate, high) was evaluated by two independent reviewers using a modified version of the Joanna Briggs Institute checklist [20, 21]. Further details on extraction methods can be found in a previous publication [22].

### 2.4 Reporting Guideline Adherence Scoring System

The ROSES‐S guideline scoring sheet split each of the 22 checklist items into their component paragraphs, with each paragraph containing specific and related reporting criteria (Data S2). As a result, a total of 68 items were scored independently. Only the second criterion of item 5 (“Describe the timing of the biological sampling in relation to the disease epidemiology in the study population [the beginning, peak, and end of virus transmission], Describe any vaccination efforts that have been undertaken”) was further split into two items (items 5.2 and 5.3), as vaccination was not relevant in the beginning of the pandemic.

To ensure inter‐rater agreement, raters (AS, BC, EL) piloted 10 eligible studies; discordances were discussed with arbitration by a co‐creator of the ROSES‐S guideline (NB). Scoring was completed by categorizing each statement as “reported” or “not reported.” A “not applicable” (N/A) option was possible for 22/68 (32.4%) items related to specific study designs or that were not relevant at all stages of the pandemic. For example, items pertaining to case‐control studies were automatically “not applicable” given that the case‐control study design was not eligible for inclusion in our study. N/A scores were not assigned a numeric value and, therefore, were not included in the denominator when calculating adherence proportions. Where there were multiple criterion embedded in a single reporting item that was not further broken down in the scoring sheet, the item was scored as “reported” if any of the criteria were satisfied. For each study, the adherence scores for each of the six domains were calculated, as well as an overall adherence score across all items.

### 2.5 Statistical Analysis

The study characteristics were summarized using descriptive statistics. We calculated the percentage of studies adhering to each item of the ROSES‐S scoring sheet, as well as the median percentage and interquartile range (IQR) of adherence across all items and for each domain (i.e., title, abstract, and introduction; epidemiological methods; laboratory methods; results; discussion; other information) and for each study characteristic (article publication source, study design, publication month, sampling frame, sampling method, sample size, overall RoB, geographic scope, WHO HRP status, WHO Unity protocol alignment, IF, and whether the study cited ROSES‐S or other reporting guidelines). Overall percentage of adherence and percentage adherence by domain were visualized with violin plots, and the relationship between IF and adherence was visually inspected using a scatter plot. A publication time lag of 154 days was used; this was the estimated median time between the last date of participant sampling and the publication date of SARS‐CoV‐2 seroepidemiologic studies during that time period, irrespective of publication type [23]. Three time periods were used: pre‐ROSES‐S publication (March 1, 2020–June 26, 2021), 0–154 days post‐ROSES‐S publication (June 27, 2021–November 27, 2021), and 155–553 days post‐ROSES‐S publication (November 28, 2021–December 31, 2022). The RoB evaluations were summated using percentages.

Beta regression was used to investigate the association of study characteristics and publication of the ROSES‐S guideline with total adherence scores. A univariable continuous piecewise model with two knots—one at the date of ROSES‐S publication and the other 154 days later—was constructed to assess the total adherence scores over time and was plotted to conduct a visual inspection of trends. A multivariable model was also built to explore all candidate study characteristic predictors. Marginal effects were calculated for the final model to facilitate the interpretation of model coefficients. All adherence scores and predicted score changes from the beta regression models are reported as percentages. See Data S2 for more details. Analysis was conducted using the betareg (version [v] 3.1.4) [24], lmtest (v 0.9.40) [25], MuMIn (v 1.47.5) [26], and mfx (v 1.2.2) [27] packages in R (v 4.2.2).

## 3 Results

**FIGURE A1:**
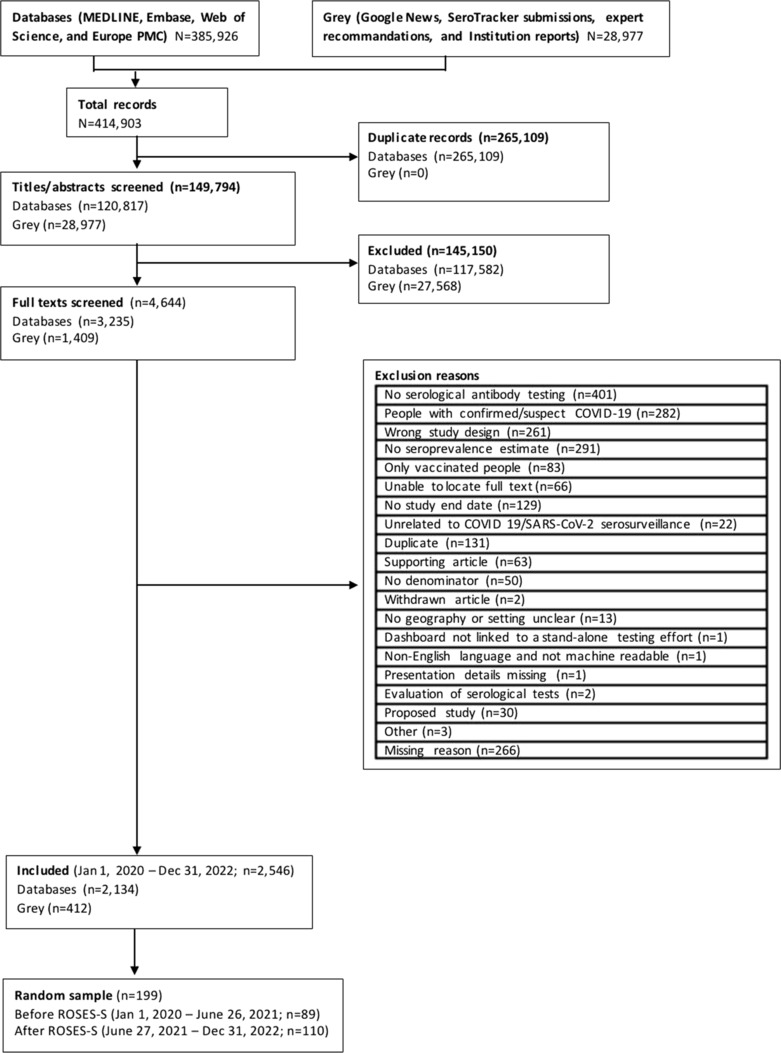
PRISMA flow diagram of inclusion.

One hundred and ninety‐nine articles were included and analyzed (Figure A1; Data S2). Study characteristics are reported in Table 1. Included studies were mostly published in peer‐reviewed journals (68.3%), used one‐time cross‐sectional survey design (74.9%), and were sampled from the general population (52.8%). The studies mostly used a non‐probability sampling method (71.3%) and incorporated a sample size of 1000+ participants (46.2%). The majority of studies were local in geographic scope (61.3%) and originated from geographic locations without HRP status (81.4%). Most studies were not aligned with the WHO Unity protocol (77.9%) and did not cite any reporting guidelines (97%), with none citing ROSES‐S. Lastly, articles were assessed to have a high RoB (61.8%), with RoB item 8b (“Was there appropriate adjustment for population characteristics?”) the least fulfilled (12.6%) (Table A1).

**TABLE 1:** Summary characteristics of included articles (*n* = 199).

Variable	Studies, *n* (%)
*N*	199
Source type, *n*/*N* (%)	
Journal article (peer‐reviewed)	136/199 (68.3)
Pre‐print	39/199 (19.6)
Institutional report	17/199 (8.5)
Conference abstract or news	7/199 (3.5)
Study design, *n*/*N* (%)	
Cross‐sectional survey	149/199 (74.9)
Repeated cross‐sectional survey	21/199 (10.6)
Cohort study	29/199 (14.6)
Sampling frame^a^, *n*/*N* (%)	
General population	105/199 (52.8)
Special population	94/199 (47.2)
Sampling method, *n*/*N* (%)	
Non‐probability	142/199 (71.3)
Probability	40/199 (20.1)
Unclear	17/199 (8.5)
Sample size, *n*/*N* (%)	
< 500	63/199 (31.7)
500–999	44/199 (22.1)
1000+	92/199 (46.2)
Overall JBI, *n*/*N* (%)	
High	123/199 (61.8)
Moderate	66/199 (33.2)
Low	10/199 (5.0)
Geographic scope, *n*/*N* (%)	
National	42/199 (21.1)
Regional	35/199 (17.6)
Local	122/199 (61.3)
HRP status, *n*/*N* (%)	
HRP	37/199 (18.6)
Non‐HRP	162/199 (81.4)
WHO Unity protocol^b^, *n*/*N* (%)	
Unity‐aligned	44/199 (22.1)
Not Unity‐aligned	155/199 (77.9)
Citation of reporting guideline, *n*/*N* (%)	
STROBE	5/199 (2.5)
TREND	1/199 (0.5)
ROSES‐S	0/199 (0)

Abbreviations: HRP, Humanitarian Response Plan; STROBE, Strengthening the Reporting of Observational Studies in Epidemiology; TREND, Transparent Reporting of Evaluations with Nonrandomized Designs.

^a^ General population includes the following sampling frames: household and community samples, blood donors, residual sera, persons living in slums, pregnant or parturient women, and representative patient populations.

^b^ WHO Unity‐aligned: studies aligned with the WHO Unity protocol.

The adherence of study reporting to ROSES‐S items is reported in Table 2. The median adherence to ROSES‐S reporting items was 48.1% (IQR 40.0%–55.2%) per study. Adherence ranged from 8.8% to 72.7% per study. Overall, the most frequently reported ROSES‐S items were “State the interval between sequential biological samples (serial cross‐sectional or longitudinal studies), or specify whether only a single sample was collected (cross‐sectional study)” (item 5.5: 197/199 [99.0%]), and “For a cross‐sectional study, report the numbers of outcome events or summary measures” (item 15.3: 160/163 [98.2%]). The least frequently reported ROSES‐S items were “For a cohort study, explain how loss to follow‐up was addressed, if applicable” (item 12.5: 0/14 [0%]) and “Specify laboratory biosafety conditions” (item 13.10): 2/198 [1.0%]).

**TABLE 2:** Adherence to ROSES‐S overall and pre/post ROSES‐S publication.

		Item		Short description^a^	Overall	ROSES‐S publication		
		Title	#			Before	0–154 days after	155–553 days after
Total *N*					199	89	50	60
All items	Median % [IQR]				48.1 [40.0, 55.2]	47.3 [38.5, 55.6]	50.9 [45.5, 55.2]	46.6 [39.6, 54.6]
Domain 1: title, abstract, and introduction	Domain total: median % [IQR]				50.0 [33.3, 60.0]	40.0 [33.3, 60.0]	50.0 [33.3, 50.0]	50.0 [33.3, 66.7]
	Item: *n*/*N* (%)	Title and abstract	1.1	Contains “seroprevalence” or “seroepidemiology”	81/197 (41.1)	32/87 (36.8)	21/50 (42.0)	28/60 (46.7)
			1.2	Structured summary	119/183 (65.0)	46/78 (59.0)	34/48 (70.8)	39/57 (68.4)
		Introduction	2.1	State antibody kinetics	39/197 (19.8)	20/87 (23.0)	9/50 (18.0)	10/60 (16.7)
			2.2	State circulating variants	42/171 (24.6)	8/62 (12.9)	8/49 (16.3)	26/60 (43.3)
			2.3	State sensitivity and specificity of assay	99/199 (49.7)	46/89 (51.7)	29/50 (58.0)	24/60 (40.0)
			3.1	State objectives	155/199 (77.9)	66/89 (74.2)	43/50 (86.0)	46/60 (76.7)
Domain 2: epidemiological methods	Domain total: median % [IQR]				50.0 [42.3, 61.2]	50.0 [42.3, 60.9]	55.6 [48.1, 65.2]	48.1 [38.5, 57.7]
	Item: *n*/*N* (%)	Study design	4.1	Explain choice of study design	110/197 (55.8)	41/87 (47.1)	35/50 (70.0)	34/60 (56.7)
		Setting	5.1	Describe location and sampling frame	185/199 (93.0)	78/89 (87.6)	48/50 (96.0)	59/60 (98.3)
			5.2	Describe timeframe of sampling in relation to disease epidemiology	86/199 (43.2)	35/89 (39.3)	22/50 (44.0)	29/60 (48.3)
			5.3	Describe vaccination efforts	64/135 (47.4)	11/32 (34.4)	16/43 (37.2)	37/60 (61.7)
			5.4	Describe timing of sampling in individuals	28/197 (14.2)	19/87 (21.8)	9/50 (18.0)	0/60 (0.0)
			5.5	State sampling interval	197/199 (99.0)	88/89 (98.9)	49/50 (98.0)	60/60 (100.0)
		Participants	6.1	Describe case ascertainment method	0/0	0/0	0/0	0/0
			6.2	For household studies, provide definitions	0/0	0/0	0/0	0/0
			6.3	For outbreak studies, describe source of cases	1/1 (100.0)	0/0	1/1 (100.0)	0/0
			6.4	For cohort studies, state eligibility criteria and sampling method	30/32 (93.8)	13/13 (100.0)	11/11 (100.0)	6/8 (75.0)
			6.5	For case‐control studies, state eligibility criteria and selection of cases/controls	0/0	0/0	0/0	0/0
			6.6	For cross‐sectional studies, state eligibility criteria and selection	154/170 (90.6)	67/78 (85.9)	39/40 (97.5)	48/52 (92.3)
		Variables	7.1	Describe outcomes, exposures, confounders	125/199 (62.8)	56/89 (62.9)	30/50 (60.0)	39/60 (65.0)
			7.2	State median age and range of exposure group	64/197 (32.5)	29/88 (33.0)	16/49 (32.7)	19/60 (31.7)
			7.3	Describe vaccination status of participants	35/151 (23.2)	7/47 (14.9)	11/44 (25.0)	17/60 (28.3)
			7.4	Describe any cross‐reactivity	27/199 (13.6)	16/89 (18.0)	5/50 (10.0)	6/60 (10.0)
			7.5	Describe illness definitions	110/196 (56.1)	56/86 (65.1)	30/50 (60.0)	24/60 (40.0)
		Data sources/measurement biases	8.1	Describe sources of data for each variable	188/199 (94.5)	84/89 (94.4)	49/50 (98.0)	55/60 (91.7)
			8.2	Describe cases and controls separately	28/67 (41.8)	15/37 (40.5)	7/15 (46.7)	6/15 (40.0)


		Bias	9.1	Describe methods to control bias	71/196 (36.2)	23/87 (26.4)	22/49 (44.9)	26/60 (43.3)
		Study size	10.1	Describe baseline seroprevalence	103/196 (52.6)	47/87 (54.0)	29/49 (59.2)	27/60 (45.0)
			10.2	Explain sample size	113/199 (56.8)	41/89 (46.1)	40/50 (80.0)	32/60 (53.3)
		Quantitative variables	11.1	Explain quantitative analyses	174/199 (87.4)	76/89 (85.4)	48/50 (96.0)	50/60 (83.3)
			11.2	Describe assay limit and its calculation	79/198 (39.9)	41/88 (46.6)	15/50 (30.0)	23/60 (38.3)
			11.3	Define seropositivity	68/196 (34.7)	33/87 (37.9)	18/49 (36.7)	17/60 (28.3)
		Statistical methods	12.1	Describe statistical methods	161/197 (81.7)	70/87 (80.5)	46/50 (92.0)	45/60 (75.0)
			12.2	Describe subgroup analyses	88/185 (47.6)	44/76 (57.9)	31/49 (63.3)	13/60 (21.7)
			12.3	Describe methods to address sampling and selection bias	64/197 (32.5)	22/87 (25.3)	21/50 (42.0)	21/60 (35.0)
			12.4	Explain methods addressing missing data	39/199 (19.6)	13/89 (14.6)	14/50 (28.0)	12/60 (20.0)
			12.5	For cohort studies, explain methods addressing loss to follow‐up	0/14 (0.0)	0/3 (0.0)	0/4 (0.0)	0/7 (0.0)
			12.6	For case‐control studies, explain matching	0/0	0/0	0/0	0/0
			12.7	For cross‐sectional studies, describe methods accounting for sampling strategy	56/158 (35.4)	13/75 (17.3)	12/32 (37.5)	31/51 (60.8)
12.8			Describe any sensitivity analyses	10/107 (9.3)	3/21 (14.3)	3/27 (11.1)	4/59 (6.8)
12.9	State any adjustments for assay performance		31/197 (15.7)	17/88 (19.3)	7/49 (14.3)	7/60 (11.7)
Domain 3: laboratory methods	Domain total: median % [IQR]				33.3 [25.0, 41.7]	33.3 [18.2, 50.0]	30.3 [25.0, 39.6]	33.3 [25.0, 41.7]
	Item: *n*/*N* (%)	Sample type and handling	13.1	Describe blood type	144/199 (72.4)	59/89 (66.3)	38/50 (76.0)	47/60 (78.3)
			13.2	Describe specimen storage	35/199 (17.6)	15/89 (16.9)	12/50 (24.0)	8/60 (13.3)
		Serological assays	13.3	Use lab methods available in more than one country	167/199 (83.9)	72/89 (80.9)	42/50 (84.0)	53/60 (88.3)
			13.4	State testing algorithm	152/199 (76.4)	67/89 (75.3)	41/50 (82.0)	44/60 (73.3)
			13.5	Reference previous protocol and describe cut‐off determination	66/198 (33.3)	30/89 (33.7)	10/49 (20.4)	26/60 (43.3)
			13.6	State determinants of assay detection variability	37/197 (18.8)	17/88 (19.3)	7/49 (14.3)	13/60 (21.7)
			13.7	Specify antigen, antibody isotope target, and how cut‐off was determined	116/196 (59.2)	51/88 (58.0)	18/48 (37.5)	47/60 (78.3)
			13.8	Describe controls	26/198 (13.1)	19/89 (21.3)	4/49 (8.2)	3/60 (5.0)
			13.9	Describe starting and end dilutions	26/198 (13.1)	13/89 (14.6)	3/49 (6.1)	10/60 (16.7)
			13.10	Specify lab biosafety conditions	2/198 (1.0)	2/89 (2.2)	0/49 (0.0)	0/60 (0.0)
			13.11	Specify any replication	12/197 (6.1)	10/88 (11.4)	2/49 (4.1)	0/60 (0.0)
			13.12	Specify any confirmatory assays	31/198 (15.7)	18/89 (20.2)	6/49 (12.2)	7/60 (11.7)
Domain 4: results	Domain total: median % [IQR]				37.5 [28.6, 50.0]	37.5 [25.0, 50.0]	42.9 [28.6, 50.0]	37.5 [25.0, 50.0]
	Item: *n*/*N* (%)	Participants	13.13	Report eligible individuals	110/197 (55.8)	42/89 (47.2)	37/48 (77.1)	31/60 (51.7)
			13.14	Explain reasons for non‐participation	20/194 (10.3)	9/85 (10.6)	7/49 (14.3)	4/60 (6.7)
		Descriptive data	14.1	Describe study participants	134/199 (67.3)	57/89 (64.0)	41/50 (82.0)	36/60 (60.0)
			14.2	Indicate number of participants with missing data	31/150 (20.7)	11/65 (16.9)	7/32 (21.9)	13/53 (24.5)
			14.3	For cohort studies, state follow‐up time	13/18 (72.2)	5/6 (83.3)	3/4 (75.0)	5/8 (62.5)
		Outcome data	15.1	For cohort studies, report number of outcome events	9/24 (37.5)	2/9 (22.2)	3/9 (33.3)	4/6 (66.7)
			15.2	For case‐control studies, report summary measures of exposure	0/0	0/0	0/0	0/0
			15.3	For cross‐sectional studies, report numbers of outcome events	160/163 (98.2)	78/78 (100.0)	33/34 (97.1)	49/51 (96.1)
		Main result	16.1	Report crude estimates by age	53/199 (26.6)	21/89 (23.6)	9/50 (18.0)	23/60 (38.3)
			16.2	Standardize results to target population	20/199 (10.1)	8/89 (9.0)	4/50 (8.0)	8/60 (13.3)
		Other analyses	17.1	Report other sub‐analyses	79/194 (40.7)	35/85 (41.2)	21/50 (42.0)	23/59 (39.0)
Domain 5: discussion	Domain total: median % [IQR]				75.0 [50.0, 100.0]	75.0 [50.0, 100.0]	100.0 [50.0, 100.0]	75.0 [50.0, 100.0]
	Item: *n*/*N* (%)	Key results	18.1	Summarize results	188/199 (94.5)	80/89 (89.9)	49/50 (98.0)	59/60 (98.3)
		Limitations	19.1	Discuss limits and strengths	143/197 (72.6)	58/88 (65.9)	40/49 (81.6)	45/60 (75.0)
		Interpretation	20.1	Interpret results in light of sources of bias	101/199 (50.8)	49/89 (55.1)	31/50 (62.0)	21/60 (35.0)
		Generalizability	21.1	Discuss generalizability	157/197 (79.7)	67/88 (76.1)	35/49 (71.4)	55/60 (91.7)
Domain 6: other information	Domain total: median % [IQR]				100.0 [0.0, 100.0]	100.0 [0.0, 100.0]	100.0 [100.0, 100.0]	100.0 [100.0, 100.0]
	Item: *n*/*N* (%)	Ethics approval	22.1	State if institutional approval was received	134/197 (68.0)	48/87 (55.2)	39/50 (78.0)	47/60 (78.3)

*Note:* Color key, based on adherence (%): 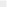 not a number; 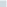 0.0–19.9; 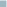 20.0–39.9; 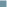 40.0–59.9; 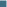 60.0–79.9; 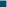 80.0–100.0.

^a^ For full description of each item, refer to Data S2.

**FIGURE A2:**
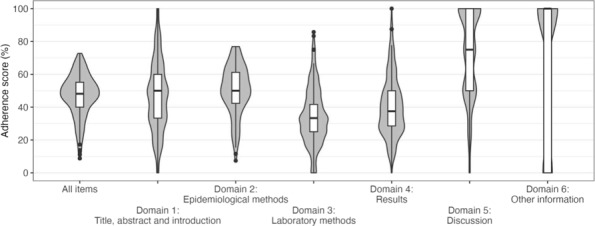
Adherence to ROSES‐S (%) overall and per domain. For all violin plots, *n* = 199, except for Domain 6, for which *n* = 197.

Adherence scores for the ROSES‐S domains varied (Table 2 and Figure A2). “Laboratory methods” (Domain 3) was the domain with the least frequently reported item(s) with a median of 33.3% (IQR 25.0%–41.7%), followed by “Results” (Domain 4) (37.5% [IQR 28.6%–50.0%]) (Table 2). Discussion (Domain 5) was considered the domain with the most frequently reported item(s), with a median of 75.0% (IQR 50.0%–100.0%). “Other information” (Domain 6) was reported in 100% of studies but only consisted of one item (item 22.1).

Reporting varied by study characteristics (Table A2). The highest median adherence to the ROSES‐S items was among pre‐print publications (52.6% [IQR 45.9%–61.5%]) and studies that used a cohort study design (50.9% [IQR 43.9%–56.1%). Studies that sampled from special populations (49.1% [IQR 43.9%–56.3%]), used a probability sampling method (51.3% [IQR 45.6%–59.6%]), incorporated a sample size of 1000+ participants (50% [IQR 43.6%–56.4%]), were assessed to have a moderate RoB (52.7% [IQR 45.3%–59.4%]), were regional in geographic scope (51.9% [IQR 43.9%–61.3%]), and originated from geographic locations with HRP status (51.9% [IQR 42.9%–56.1%]) had the highest median adherence to the ROSES‐S items. Studies that were aligned with the WHO Unity protocol (49.1% [IQR 42.8%–53.7%]) and cited any reporting guideline (STROBE and TREND) (57% [IQR 48.9%–59.2%]) also had the highest median adherence to the ROSES‐S items.

**FIGURE 1:**
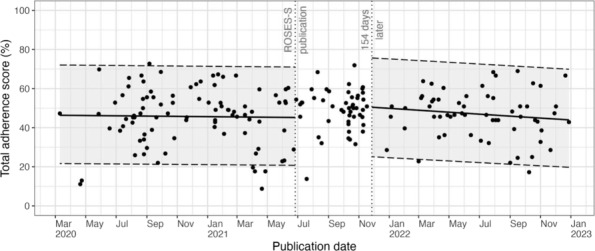
Adherence to ROSES‐S over time. Each dot represents a study. Dashed lines are the 2.5% and 97.5% quantiles of the predicted beta distribution. This piecewise model was not statistically significant compared to non‐segmented [X^2^ (2) = 2.68, *p* = 0.262] and null [X^2^ (3) = 2.85, *p* = 0.415] models in likelihood ratio tests.

Median adherence to the ROSES‐S items was similar before and after the publication of the ROSES‐S guideline (Table 2). Median adherence was 47.3% (IQR 38.5%–55.6%) prior to ROSES‐S publication, 50.9% (IQR 45.5%–55.2%) 0–154 days after publication, and 46.6% (IQR 39.6%–54.6%) 155–553 days after publication. Adherence to items fluctuated over time, increasing for some items after the publication of the ROSES‐S guideline and decreasing for others. For example, reporting of “For a cross‐sectional study, describe analytical methods taking account of sampling strategy, if applicable” (item 12.7) increased from 17.3% (13/75 studies) pre‐ROSES‐S to 60.8% (31/51 studies) 155–553 days post‐ROSES‐S. In contrast, reporting of “Describe illness definitions and methods for ascertaining the presence or absence of clinical illness in subjects” (item 7.5) decreased from 65.1% (56/86 studies) pre‐ROSES‐S to 40% (24/60 studies) 155–553 days post‐ROSES‐S. Piecewise time series analysis showed no significant changes in reporting after the publication of the ROSES‐S guideline (Figure 1).

Multivariable analysis for beta regression is presented in Table 3. Overall reporting adherence was significantly associated with article publication source, study RoB, and sampling method. Institutional reports and conference abstracts or news articles had considerably lower mean total adherence to ROSES‐S compared to published peer‐reviewed journal articles, respectively, at −14.0% (−19.4% to −8.6%) and −29% (−35.1% to −22.9%). Mean total adherence to ROSES‐S was 5.8% (2.3%–9.3%) and 7.8% (0.3%–15.2%) higher for studies with moderate and low RoB, respectively, compared to those with a high RoB. Furthermore, studies that used an unclear sampling method had a −9.4% (15.8% to −2.9%) lower mean total adherence to ROSES‐S compared to those that used a non‐probability sampling method. Univariable beta regression also showed that special population studies had higher adherence scores compared to general population studies with an increase of 4.2% (0.5%–7.8%) (Table A3).

**TABLE 3:** Multivariable beta regression identifying factors associated with reporting completeness.

Component	Variable		Model coefficients			Marginal effects (%)	
			Estimate (95% CI)	*p*		Estimate (95% CI)	*p*
Mean	(Intercept)	(Intercept)	−0.12 (−0.21 to −0.04)	0.006**			
	Source type	Peer‐reviewed journal article	Reference			Reference	
		Pre‐print	0.14 (0.00–0.28)	0.051		3.54 (−0.04–7.12)	0.051
		Institutional report	−0.58 (−0.82 to −0.34)	< 0.001***		−13.98 (−19.41 to −8.55)	< 0.001***
		Conference abstract or news	−1.37 (−1.76 to −0.99)	< 0.001***		−29.03 (−35.13 to −22.94)	< 0.001***
	Overall risk of bias	High	Reference			Reference	
		Moderate	0.23 (0.09–0.37)	0.001**		5.81 (2.32–9.31)	0.001**
		Low	0.31 (0.01–0.61)	0.041*		7.77 (0.32–15.22)	0.040*
	Sampling method	Non‐probability	Reference			Reference	
		Probability	0.00 (−0.15–0.16)	0.955		0.11 (−3.86‐4.08)	0.955
		Unclear	−0.38 (−0.66 to −0.11)	0.006**		−9.37 (−15.84 to −2.90)	0.004**
Precision	(Intercept)	(Intercept)	3.05 (−0.21 to −0.04)	< 0.001***			
	Sampling method	Non‐probability	Reference				
		Probability	0.56 (−0.15–0.16)	0.025*			
		Unclear	−0.38 (−0.66 to −0.11)	0.277			

* *p* < 0.05,

** *p* < 0.01, and

*** *p* < 0.001.

**FIGURE A3:**
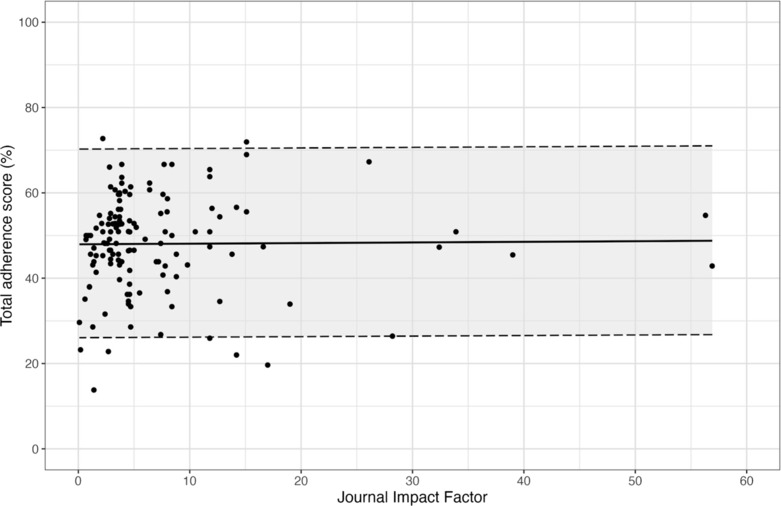
Association between the adherence score of a study and its publisher's journal impact factor. This plot only shows the included studies published in a peer‐reviewed journal with an impact factor (*N* = 133). Each dot represents a study. Dashed lines are the 2.5% and 97.5% quantiles of the predicted beta distribution.

Lastly, IF and adherence score were plotted for included peer‐reviewed studies with available IFs (*N* = 133) to assess whether there was an association between quality of manuscript and adherence score, using IF as a proxy for quality (Figure A3). The mean IF across studies was 7.18. Upon visual inspection, there did not appear to be a relationship between IF and adherence score. The fitted univariable beta regression did not show a significant change in average adherence score with one unit increase in IF [0.01% (−0.21%–0.23%)] (Table A3).

## 4 Discussion

The ROSES‐S guideline was published to facilitate the reporting of a minimal collection of information in SARS‐CoV‐2 seroepidemiologic studies. This systematic review sub‐study examined the completeness and associated factors of reporting in SARS‐CoV‐2 seroepidemiologic studies published in the first two years of the COVID‐19 pandemic as determined by adherence to the ROSES‐S guideline. Reporting completeness was found to be suboptimal, which may have implications for consumers of said scientific literature in assessing study quality. The median adherence to ROSES‐S items was 48.1% per study, which is comparable to estimates from studies evaluating adherence to STROBE and STROME‐ID [7, 8, 9, 10, 11].

There was variation in the completeness of reporting for different reporting items. The domains of the ROSES‐S guideline with the least frequently reported items were laboratory methods and results, and the least reported individual items were related to serological assays and statistical methods. This aligns with evaluations of STROBE, where key methodological items seem to be consistently underreported (e.g., sample size estimation, addressing missing data, addressing loss to follow‐up) [28]. This trend persists in evidence synthesis citing more heavily journal endorsed and established guidelines, such as the PRISMA statement [29], with one review finding fewer than 67% of systematic reviews adhering to items relating to protocol and registration, search, data items, RoB across and within studies, and additional analyses [30].

The publication of the ROSES‐S guideline was not associated with a change in reporting completeness, suggesting that the guideline has not had an impact on reporting practices to date. Other studies using segmented regression to evaluate the impact of the STROBE guidelines publication on reporting have similarly found no significant association [8]. This contrasts with evaluations of the “Consolidated Standards of Reporting Trials” (CONSORT) guideline [31], which was originally published in 1996 and last updated in 2001 [32], as well as the PRISMA statement. According to PRISMA, from publication in 2010 to 2016, item reporting adherence increased in systematic reviews, compared to systematic reviews published from 1989 to 2016 [30]. This suggests that a longer follow‐up time may be needed to increase awareness and uptake of the ROSES‐S guideline, which was published in 2021. This is reinforced by our finding that only 3.7% of peer‐reviewed studies cited a reporting guideline, none of which were ROSES‐S, suggesting a lack of awareness of the ROSES‐S guideline.

Complete reporting of methodological items is necessary to inform appropriate adjustments of estimates based on differences in study methodology. Methodological heterogeneity has been observed in previous syntheses of SARS‐CoV‐2 seroepidemiologic studies and has been associated with notable differences in estimates [30, 31]. For example, in one study, failing to account for methodological differences between serosurveys led to variation in estimates as high as 16% [31]. In instances where methods are not clearly reported, adjustment may not be possible, and this may affect the accuracy, comparability, and generalizability of synthesized seroprevalence estimates.

Complete reporting of items related to sampling and measurement methods is particularly important given their impact on study RoB [21]. ROSES‐S items, such as those related to sample recruitment, size, and retention, as well as immunoassay validation and testing algorithm, inform judgments of selection bias and measurement bias, respectively [21]. For instance, incomplete reporting of immunoassay validation details may contribute to misleading results, as it limits the opportunity for adjustment of seroprevalence estimates for poor test performance, which has been shown to heavily influence seroprevalence estimates [31]. Incomplete reporting thus directly affects RoB assessment and creates the potential for misleading meta‐analyzed estimates, hindering the utility of individual studies and evidence synthesis.

Journal IF is an important metric to assess the visibility and reputation of a journal. It was used as a proxy for quality to investigate the association between quality of manuscript and adherence score. Because the results of this study did not demonstrate that IF had any relationship with or influence on adherence score, it can be deduced that IF does not reflect the reporting completeness of individual studies.

Interventions to improve adherence to reporting guidelines have been proposed and tested. For instance, there could be greater encouragement of use of the guidelines, such as integrating checklist writing tools in the submission process (e.g., COBWEB), which has improved overall reporting completeness for randomized controlled trials [33]. Although journal endorsement of reporting guidelines seems to impact adherence [6, 32, 34], it can be ineffective without enforcement. Verifying adherence to a guideline throughout the peer‐review process could also be considered. Editors may ask authors to flag nonadherence to a given guideline. When such an intervention was evaluated using the CONSORT guideline, improvements in reporting were demonstrated [35]. Peer‐review tools, such as StatReviewer, that automatically check guideline adherence against manuscripts may also improve adherence [36].

There are other potential solutions specifically tailored to the reporting of serosurveillance data. For instance, reporting guidelines could be included in epidemiological study protocols, like the Unity protocol for COVID‐19 [19], to increase awareness, and to place emphasis on methodological quality and reporting completeness at the outset of conducting a study. Reporting completeness and guidelines should also be considered by stakeholders establishing and maintaining serological surveillance networks in preparation for the next pandemic.

We found that institutional reports, conference abstracts, and news and media reports had lower ROSES‐S adherence compared to peer‐reviewed journal articles. This may in part be attributable to limited reporting space in shorter form publication outlets like media reports and conference abstracts. Although the ROSES‐S guideline was designed for full length seroepidemiologic study articles, gray literature including news media or public health reports can be used in evidence synthesis and, therefore, may warrant development of an adapted guideline for short form articles. Meanwhile, the lack of publication standards and peer‐review for institutional reports may be contributing factors for poor reporting. The existing ROSES‐S guideline is a suitable resource for those types of articles and could be better utilized.

Our results showed that RoB was associated with adherence to the ROSES‐S guideline, similar to other studies that have found correlations between lower RoB and reporting completeness [37, 38]. This association may arise from differences in the behaviors, traits, or circumstances of investigators that produce studies of higher versus lower methodological quality. For example, investigators conducting studies of higher methodological quality may have more resources and time to produce comprehensive studies, or more training and knowledge about study methods, including reporting. If so, enhanced training or additional resources for investigators conducting seroepidemiologic studies may be needed to enable comprehensive reporting (e.g., user‐friendly templates).

This study had two key strengths. Firstly, to the best of our knowledge, this was the first analysis to evaluate the reporting completeness of SARS‐CoV‐2 seroepidemiologic studies according to the ROSES‐S guideline. Secondly, we sampled from the largest global repository of SARS‐CoV‐2 seroepidemiologic studies that was developed using a robust systematic review; thus, the results likely have good generalizability.

This study had several limitations. Firstly, the 154‐day median publication delay time utilized in the analysis may not have been sufficiently long. Articles published after December 2021 may have been submitted before publication of the ROSES‐S guideline and, therefore, subject to misclassification bias as the guideline was not available when the authors were reporting their data. However, our sensitivity analysis using a longer time delay (255 days, the third quartile of previously cited median publication lag time) [23] did not differ from the main analysis (Table A3). Longer delays to publication are possible, but likely only affected a small number of studies [23]. Secondly, as some reporting criterion needed to be reported together to be interpretable, we may have overestimated adherence to multi‐conditional items given that we scored the item as “reported” if any criterion were satisfied. Thirdly, decisions on whether a reporting item was achieved were subjective and other investigators may evaluate adherence differently. However, we mitigated this by developing our scoring sheet items in consultation with developers of the ROSES‐S guideline, piloted our scoring sheet, and discussed discrepancies to ensure inter‐rater agreement.

Examining study quality is an aim for our future work, building upon the knowledge gained through this review and others, and would consider the influence of different variables before classifying a manuscript as either good or bad quality.

In summary, this study found that the completeness of reporting in SARS‐CoV‐2 seroepidemiologic studies was suboptimal and associated with key study characteristics including article source of publication, RoB, and sampling method. The publication of the ROSES‐S guideline was not associated with changes in reporting practices. Improvements in the completeness of reporting may serve to increase the transparency, comparability, and reproducibility of seroepidemiologic research. Authors, journal editors, funders, decision‐makers, and other stakeholders in infectious disease research and public health should consider approaches to facilitate guideline adherence such as ROSES‐S guideline endorsement and promotion, journal policies on reporting, and other potential funding incentives.

## Author Contributions

**Brianna Cheng:** Conceptualization; Investigation; Methodology; Project administration; Supervision; Writing – original draft; Writing – review and editing. **Emma Loeschnik:** Investigation; Methodology; Project administration; Supervision; Writing – original draft; Writing – review and editing. **Anabel Selemon:** Investigation; Methodology; Project administration; Writing – original draft; Writing – review and editing. **Reza Hosseini:** Data curation; Formal analysis; Visualization; Writing – original draft; Writing – review and editing. **Jane Yuan:** Data curation; Formal analysis; Visualization; Writing – original draft; Writing – review and editing. **Harriet Ware:** Formal analysis; Visualization; Writing – original draft; Writing – review and editing. **Xiaomeng Ma:** Methodology; Writing – original draft; Writing – review and editing. **Christian Cao:** Data curation; Writing – original draft; Writing – review and editing. **Isabel Bergeri:** Conceptualization; Methodology; Writing – original draft; Writing – review and editing. **Lorenzo Subissi:** Methodology; Writing – original draft; Writing – review and editing. **Hannah C. Lewis:** Methodology; Writing – original draft; Writing – review and editing. **Tyler Williamson:** Supervision; Writing – review and editing. **Paul Ronksley:** Supervision; Writing – review and editing. **Rahul K. Arora:** Conceptualization; Funding acquisition; Methodology; Project administration; Writing – review and editing. **Mairead Whelan:** Funding acquisition; Project administration; Resources; Supervision; Writing – original draft; Writing – review and editing. **Niklas Bobrovitz:** Conceptualization; Funding acquisition; Methodology; Project administration; Supervision; Writing – original draft; Writing – review and editing.

## Ethics Statement

This study did not require ethics approval.

## Conflicts of Interest

The authors declare no conflicts of interest.

### Peer Review

The peer review history for this article is available at https://www.webofscience.com/api/gateway/wos/peer‐review/10.1111/irv.13283.

## Supporting information


**Data S1** Supporting Information

**Data S2** Supporting Information

## Data Availability

Findings of this study are available within the article and its supplementary materials. The corresponding author may be contacted for all other requests.

## Appendix A

**TABLE A1:** Risk of bias of included studies based on the modified version of the Joanna Briggs Institute (JBI) checklist.

Variable	Description	Overall	ROSES‐S publication		
			Before	0–154 days after	155–553 days after
*N*		199	89	50	60
Overall risk of bias *n*/*N* (%)					
High		123/199 (61.8)	61/89 (68.5)	26/50 (52.0)	36/60 (60.0)
Moderate		66/199 (33.2)	25/89 (28.1)	22/50 (44.0)	19/60 (31.7)
Low		10/199 (5.0)	3/89 (3.4)	2/50 (4.0)	5/60 (8.3)
Modified JBI item = yes *n*/*N* (%)					
JBI 1	Was the sample frame appropriate to address the target population?	85/199 (42.7)	36/89 (40.4)	21/50 (42.0)	28/60 (46.7)
JBI 2	Were study participants recruited in an appropriate way?	40/199 (20.1)	14/89 (15.7)	10/50 (20.0)	16/60 (26.7)
JBI 3	Was the sample size adequate?	125/199 (62.8)	57/89 (64.0)	35/50 (70.0)	33/60 (55.0)
JBI 4	Were the study subjects and the setting described in detail?	121/199 (60.8)	47/89 (52.8)	41/50 (82.0)	33/60 (55.0)
JBI 5	Was data analysis conducted with sufficient coverage of the identified sample?	60/199 (30.2)	29/89 (32.6)	12/50 (24.0)	19/60 (31.7)
JBI 6	Were valid methods used for the identification of the condition?	102/199 (51.3)	44/89 (49.4)	25/50 (50.0)	33/60 (55.0)
JBI 7	Was the condition measured in a standard, reliable way for all participants?	199/199 (100.0)	89/89 (100.0)	50/50 (100.0)	60/60 (100.0)
JBI 8a	Was there appropriate adjustment for test characteristics?	110/199 (55.3)	50/89 (56.2)	27/50 (54.0)	33/60 (55.0)
JBI 8b	Was there appropriate adjustment for population characteristics?	25/199 (12.6)	9/89 (10.1)	6/50 (12.0)	10/60 (16.7)
JBI 9	Was the response rate adequate, and if not, was the low response rate unlikely to introduce bias?	37/199 (18.6)	18/89 (20.2)	15/50 (30.0)	4/60 (6.7)

*Note:* Color key, based on percentage of item being “yes”:  0.0–19.9;  20.0–39.9;  40.0–59.9;  60.0–79.9;  80.0–100.0.

**TABLE A2:** Median and IQR adherence scores of study characteristics stratified by ROSES‐S publication time period.

Variable			Overall		ROSES‐S publication				
					Before	0–154 days after		155–553 days after	
		*n* (%)	Median [IQR]	*n* (%)	Median [IQR]	*n* (%)	Median [IQR]	*n* (%)	Median [IQR]
Source type									
Journal article (peer‐reviewed)		136 (68.3)	49.1 [43.0, 54.8]	57 (28.6)	48.1 [40.4, 55.6]	41 (20.6)	50.9 [43.4, 54.4]	38 (19.1)	47.4 [43.9, 53.2]
Pre‐print		39 (19.6)	52.6 [45.9, 61.5]	18 (9.0)	51.8 [46.6, 60.5]	7 (3.5)	55.2 [47.4, 56.7]	14 (7.0)	53 [45.6, 62.7]
Institutional report		17 (8.5)	38.6 [28.8, 45.3]	12 (6.0)	33.8 [24.5, 40.4]	2 (1.0)	48.1 [47.2, 49.1]	3 (1.5)	38.6 [35.4, 46.5]
Conference abstract or news		7 (3.5)	22 [15.1, 23.7]	2 (1.0)	12 [11.6, 12.5]	—	—	5 (2.5)	22.8 [22.0, 24.6]
Study design									
Cross‐sectional survey		149 (74.9)	47.3 [38.0, 55.6]	72 (36.2)	46.8 [34.5, 55.8]	37 (18.6)	51.7 [45.5, 56.4]	40 (20.1)	45.6 [36.2, 52.8]
Repeated cross‐sectional survey		21 (10.6)	47.4 [45.6, 53.4]	7 (3.5)	46.4 [39.3, 50.4]	2 (1.0)	48.1 [47.2, 49.1]	12 (6.0)	49.6 [46.2, 54.4]
Cohort study		29 (14.6)	50.9 [43.9, 56.1]	10 (5.0)	52.8 [46.2, 60.4]	11 (5.5)	50.8 [41.0, 53.1]	8 (4.0)	51.3 [43.9, 57.8]
Sampling frame									
General population^a^		105 (52.8)	47.1 [38.0, 54.4]	43 (21.6)	44.4 [27.8, 52.2]	18 (9.0)	57.8 [47.2, 56.2]	44 (22.1)	47.4 [39.1, 54.6]
Special population		94 (47.2)	49.1 [43.9, 56.3]	46 (23.1)	50.9 [44.0, 59.6]	32 (16.1)	49.5 [43.3, 54.6]	16 (8.0)	46.6 [43.9, 54.8]
Sampling method									
Non‐probability		142 (71.3)	48.1 [42.7, 55.1]	66 (33.2)	47.7 [42.7, 56.6]	36 (18.1)	50.4 [44.9, 54.6]	40 (20.1)	46.1 [39.4, 52.8]
Probability		40 (20.1)	51.3 [45.6, 59.6]	14 (7.0)	49.1 [38.6, 55.3]	10 (5.0)	52.3 [50.2, 59.2]	16 (8.0)	50.9 [45.8, 61.7]
Unclear		17 (8.5)	34.5 [22.0, 50.0]	9 (4.5)	23.2 [17.6, 34.6]	4 (2.0)	46.7 [41.2, 51.6]	4 (2.0)	31.8 [26.9, 40.4]
Sample size									
< 500		63 (31.7)	46.6 [39.5, 52.8]	28 (14.1)	47.7 [39.9, 52.7]	17 (8.5)	48.1 [41.4, 52.6]	18 (9.0)	44.7 [37.9, 55.0]
500–999		44 (22.1)	46.5 [35.0, 56.7]	20 (10.1)	45.9 [32.7, 59.8]	10 (5.0)	51.4 [40.1, 55.0]	14 (7.0)	46.5 [35.4, 54.8]
1000+		92 (46.2)	50 [43.6, 56.4]	41 (20.6)	48.1 [38.9, 56.6]	23 (11.6)	51.7 [48.6, 57.1]	28 (14.1)	49.1 [43.6, 53.7]
Overall JBI									
High		123 (61.8)	46.6 [36.9, 52.8]	61 (30.7)	46.6 [36.5, 52.8]	26 (13.1)	48.6 [42.1, 52.5]	36 (18.1)	45.6 [35.9, 51.3]
Moderate		66 (33.2)	52.7 [45.3, 59.4]	25 (12.6)	52.8 [42.9, 59.6]	22 (11.1)	54.8 [49.3, 59.4]	19 (9.5)	50.9 [43.4, 54.8]
Low		10 (5.0)	48.1 [38.6, 58.6]	3 (1.5)	38.5 [32.6, 38.7]	2 (1.0)	48.1 [47.2, 49.1]	5 (2.5)	61.4 [50.0, 62.5]
Geographic scope									
National		42 (21.1)	49.1 [39.1, 52.8]	17 (8.5)	39.6 [36.8, 52.6]	11 (5.5)	50.9 [48.1, 54.0]	14 (7.0)	50.4 [46.1, 53.2]
Regional		35 (17.6)	51.9 [43.9, 61.3]	12 (6.0)	58.5 [51.4, 65.6]	8 (4.0)	53.2 [50.4, 60.2]	15 (7.5)	43.9 [33.0, 51.8]
Local		122 (61.3)	47.3 [40.6, 55.1]	60 (30.2)	47.2 [37.1, 53.3]	31 (15.6)	49.1 [42.5, 54.8]	31 (15.6)	46.6 [41.4, 55.2]
HRP status									
HRP		37 (18.6)	51.9 [42.9, 56.1]	14 (7.0)	44 [41.0, 54.2]	5 (2.5)	56.4 [52.8, 59.6]	18 (9.0)	51.3 [43.1, 55.9]
Non‐HRP		162 (81.4)	47.8 [39.6, 55.0]	75 (37.7)	44 [41.0, 54.2]	45 (22.6)	56.4 [52.8, 59.6]	42 (21.1)	51.3 [43.1, 55.9]
WHO Unity protocol^b^									
Unity‐aligned		44 (22.1)	49.1 [42.8, 53.7]	18 (9.0)	44.9 [38.6, 47.9]	12 (6.0)	51.9 [49.1, 59.7]	14 (7.0)	50.9 [46.4, 54.4]
Not Unity‐aligned		155 (77.9)	48.1 [39.5, 55.6]	71 (35.7	49.1 [37.9, 57.5]	38 (19.1)	50.4 [43.9, 55.0]	46 (23.1)	45.6 [36.8, 55.0]
Citation of reporting guideline									
Yes		6 (3.0)	57 [48.9, 59.2]	2 (1.0)	63.5 [61.6, 65.4]	1 (0.5)	57.9 [57.9, 57.9]	3 (1.5)	46.6 [46.1, 51.3]
	STROBE	5 (2.5)	57.9 [56.1, 59.6]	2 (1.0)	63.5 [61.6, 65.4]	1 (0.5)	57.9 [57.9, 57.9]	2 (1.0)	51.3 [48.9, 53.7]
	TREND	1 (0.5)	45.6 [45.6, 45.6]	—	—	—	—	1 (0.5)	45.6 [45.6, 45.6]
No		193 (97.0)	48.1 [39.6, 55.2]	87 (43.7)	47.1 [37.9, 55.1]	49 (24.6)	50.8 [45.5, 55.2]	57 (28.6)	46.6 [39.3, 54.4]

Abbreviations: HRP, Humanitarian Response Plan; STROBE, Strengthening the Reporting of Observational Studies in Epidemiology; TREND, Transparent Reporting of Evaluations with Nonrandomized Designs.

^a^ General population includes the following sampling frames: household and community samples, blood donors, residual sera, persons living in slums, pregnant or parturient women, and representative patient populations.

^b^ WHO Unity‐aligned: studies aligned with the WHO Unity protocol.

**TABLE A3:** Univariable beta regression separately evaluating the association of each candidate predictor with total adherence score.

Variable		Model coefficients		Marginal effects (%)	
		Estimate (95% CI)	*p*	Estimate (95% CI)	*p*
Publication date, compared to ROSES‐S (month); 154‐day lag^a^	(Intercept)	49.58 (−409.43–508.59)			
	Before	0.00 (−0.03–0.02)	0.815	−0.07 (−0.69‐0.54)	0.815
	0–154 days after	0.04 (−0.03–0.12)	0.256	1.11 (−0.82–3.03)	0.256
	155–553 days after	−0.06 (−0.14–0.02)	0.126	−1.53 (−3.51–0.44)	0.126
Publication date, compared to ROSES‐S (month); 255‐day lag^a^	(Intercept)	−17.12 (−443.12–408.89)			
	Before	0.00 (−0.02–0.02)	0.955	0.02 (−0.56–0.59)	0.955
	0–255 days after	0.02 (−0.03–0.07)	0.463	0.50 (−0.84–1.83)	0.463
	256–550 days after	−0.05 (−0.12–0.02)	0.166	−1.26 (−3.06–0.54)	0.166
WHO HRP status	(Intercept)	−0.15 (−0.23 to −0.07)			
	Non‐HRP	Reference		Reference	
	HRP	0.09 (−0.10–0.28)	0.351	2.23 (−2.49–6.96)	0.352
WHO Unity alignment	(Intercept)	−0.14 (−0.22 to −0.05)			
	Not Unity‐aligned	Reference		Reference	
	Unity‐aligned	0.03 (−0.14–0.21)	0.719	0.81 (−3.63–5.24)	0.720
Sampling frame	(Intercept)	−0.21 (−0.31 to −0.11)			
	General population^b^	Reference		Reference	
	Special population	0.17 (0.02–0.31)	0.024*	4.16 (0.52–7.80)	0.024*
Geographic scope	(Intercept)	−0.16 (−0.25 to −0.06)			
	Local	Reference		Reference	
	Regional	0.13 (−0.07–0.33)	0.191	3.29 (−1.68–8.25)	0.192
	National	0.01 (−0.17–0.20)	0.878	0.36 (−4.27–4.99)	0.878
Study design	(Intercept)	−0.15 (−0.24 to –0.07)			
	Cross‐sectional survey	Reference		Reference	
	Repeated cross‐sectional study	0.06 (−0.18–0.30)	0.607	1.57 (−4.47–7.61)	0.607
	Cohort study	0.12 (−0.09–0.33)	0.249	3.07 (−2.19–8.33)	0.249
Sample size	(Intercept)	−0.13 (−0.21 to −0.06)			
	Sample size	0.00 (0.00–0.00)	0.777	0.00 (0.00–0.00)	0.777
Overall risk of bias	(Intercept)	−0.23 (−0.32 to −0.14)			
	High	Reference		Reference	
	Moderate	0.27 (0.12–0.43)	< 0.001***	6.82 (2.96–10.67)	< 0.001***
	Low	0.10 (−0.23–0.43)	0.544	2.56 (−5.77–10.90)	0.544
Sampling method	(Intercept)	−0.10 (−0.18 to −0.02)			
	Non‐probability	Reference		Reference	
	Probability	0.10 (−0.08–0.27)	0.277	2.43 (−1.99‐6.85)	0.278
	Unclear	−0.58 (−0.85 to −0.32)	< 0.001***	−14.02 (−20.02 to −8.02)	< 0.001***
Source type	(Intercept)	−0.08 (−0.15–0.00)			
	Peer‐reviewed journal article	Reference		Reference	
	Preprint	0.20 (0.04–0.36)	0.012*	5.08 (1.07–9.08)	0.012*
	Institutional report	−0.55 (−0.79 to −0.32)	< 0.001***	−13.26 (−18.64 to −7.88)	< 0.001***
	Conference abstract or news	−1.28 (−1.69 to −0.88)	< 0.001***	−27.59 (−34.30 to −20.87)	< 0.001***
Citation of any guidelines^c^	(Intercept)	−0.14 (−0.21 to −0.07)			
	No	Reference		Reference	
	Yes	0.35 (−0.08–0.78)	0.108	8.76 (−1.90–19.41)	0.105
Journal impact factor^d^	(Intercept)	−0.08 (−0.18–0.02)			
	Journal impact factor	0.00 (−0.01–0.01)	0.896	0.01 (−0.21–0.23)	0.896

Abbreviation: HRP, Humanitarian Response Plan.

^a^ Publication date is a continuous variable with monthly increments. Each month is considered equal to 30.4 days.

^b^ General population includes the following sampling frames: household and community samples, blood donors, residual sera, persons living in slums, pregnant or parturient women, and representative patient populations.

^c^ None cited ROSES‐S. The only cited guidelines were STROBE (*n* = 5) and TREND (*n* = 1).

^d^ Only studies published in a peer‐reviewed journal with a journal impact factor (*n* = 133) were included in this model.

* *p* < 0.05,

** *p* < 0.01, and

*** *p* < 0.001.
