# Supplementary material for: Adherence of SARS‐CoV‐2 Seroepidemiologic Studies to the ROSES‐S Reporting Guideline During the COVID‐19 Pandemic
Source: Influenza Other Respir Viruses. 2024 Jul 25;18(7):e13283. doi: 10.1111/irv.13283 (PMC11272216; doi:10.1111/irv.13283)
Supplement: Supplementary file 2 — Data S2 Supporting Information [file IRV-18-e13283-s001.docx]

**Supplementary file 1: ROSES-S scoring sheet**

| **Domain** | **Item** | | **Description** |
| --- | --- | --- | --- |
|  | **Title** | **#** |  |
| Domain 1: Title, abstract and introduction | Title and abstract | 1.1 | The term “seroepidemiologic,” “seroepidemiology,” “seroprevalence,” or “seroincidence” should be applied to the study in the title and abstract, and the medical subject heading “Seroepidemiologic Studies” be used when the report is of a population-based serological survey. |
|  |  | 1.2 | Provide a structured summary including, as applicable: objectives; population level (ie, national, regional, local), study design, study period, eligibility criteria of study participants, sampling dates and method, sample size, laboratory methods (assay used), results: seroprevalence and 95% CI, study limitations, conclusions and implications of key findings |
|  | Introduction | 2.1 | State what is known about the kinetics of antibody rise, decay, and persistence following SARS-CoV-2 infection, in the particular study setting/population, if possible. |
|  |  | 2.2 | State which SARS-CoV-2 viruses are circulating, including any variants. |
|  |  | 2.3 | State what is known about the sensitivity and specificity of the antibody detection assay being used. |
|  |  | 3.1 | State specific objectives, including any prespecified hypotheses |
| Domain 2: Epidemiological methods | Study design | 4.1 | State which specific seroepidemiologic study design was chosen and why. |
|  | Setting | 5.1 | Describe the setting, locations, and sampling frame, including periods of recruitment, exposure, follow-up, and data collection. |
|  |  | 5.2 | Describe the timing of the biological sampling in relation to the disease epidemiology in the study population (the beginning, peak, and end of virus transmission). |
|  |  | 5.3 | Describe any vaccination efforts that have been undertaken. |
|  |  | 5.4 | Where known, describe the timing of biological sampling in individuals in relation to disease onset and to exposures of interest. |
|  |  | 5.5 | State the interval between sequential biological samples (serial cross-sectional or longitudinal studies), or specify whether only a single sample was collected (cross-sectional study). |
|  | Participants | 6.1 | For case‐ascertained transmission studies, describe the method of case ascertainment and criteria for defining a “case.” Describe methods of follow‐up. |
|  |  | 6.2 | For household‐ or institution‐based transmission studies, describe the definition of a household or the institution. Describe methods of follow‐up. |
|  |  | 6.3 | For outbreak investigations involving serologic sampling, describe the setting in which the cases were identified, for example, village/residential setting, occupational workplace. Describe methods of follow‐up. |
|  |  | 6.4 | For a cohort study, give the eligibility criteria, and the sources and methods of sampling of participants. Describe methods of follow‐up. |
|  |  | 6.5 | For a case‐control study, give the eligibility criteria, and the sources and methods of case ascertainment and control selection. Give the rationale for the choice of cases and controls. For matched studies, give matching criteria and the number of controls per case. |
|  |  | 6.6 | For a cross‐sectional study, give the eligibility criteria, and the sources and methods of selection of participants. |
|  | Variables | 7.1 | Clearly define all outcomes, exposures, predictors, potential confounders, and effect modifiers. |
|  |  | 7.2 | The median age and range for each exposure group should be reported. |
|  |  | 7.3 | Describe the vaccination status of participants (specify vaccination status, vaccine manufacturer, number of doses, and timing of vaccination in relationship to collection of serum), if applicable, to affect the outcome measures. If relevant, describe measures taken to identify and record immunization history. |
|  |  | 7.4 | Describe any known or potential immunological cross‐reactivity that may bias the outcome measures. |
|  |  | 7.5 | Describe illness definitions and methods for ascertaining the presence or absence of clinical illness in subjects. |
|  | Data sources/ measurement biases | 8.1 | For each variable of interest, give sources of data and details of methods of assessment (measurement). Describe comparability of assessment methods if there is more than one group. |
|  |  | 8.2 | Give information separately for cases and controls in case‐control studies and, if applicable, for exposed and unexposed groups in cohort and cross‐sectional studies). |
|  | Bias | 9.1 | Describe any efforts to address potential sources of bias. |
|  | Study size | 10.1 | Describe the baseline estimated seroprevalence or incidence of infection and cite published literature to support these estimates. |
|  |  | 10.2 | Explain the steps that led to the final sample size. Report the numbers of individuals at each stage of the study—the numbers potentially eligible, examined for eligibility, confirmed eligible, included in the study, completing follow‐up, and analyzed. |
|  | Quantitative variables | 11.1 | Explain how quantitative variables were handled in the analyses. If applicable, describe which groupings were chosen and why. |
|  |  | 11.2 | Describe the serological assay's limit of detection and how this limit is defined or calculated. Describe how samples with a result below or on the borderline of the limit were handled in the analysis. |
|  |  | 11.3 | Define “seropositivity,” or the antibody titer change or change in other assay result used to define “seroconversion.” Avoid the term “seroconversion” unless referring to change from undetectable to detectable antibody level. Avoid the term “infection” but report “seroprevalence at a titer of ….”. |
|  | Statistical methods | 12.1 | Describe all statistical methods, including those used to control for confounding. |
|  |  | 12.2 | Describe any methods used to examine subgroups and interactions. |
|  |  | 12.3 | Describe all methods used to address sampling and selection biases (eg, weighting results, multilevel regression and post‐stratification). |
|  |  | 12.4 | Explain how missing data were addressed. |
|  |  | 12.5 | For a cohort study, explain how loss to follow‐up was addressed, if applicable. |
|  |  | 12.6 | For a case‐control study, explain how variables on which cases and controls were matched, if applicable. |
|  |  | 12.7 | For a cross‐sectional study, describe analytical methods taking account of sampling strategy, if applicable. |
|  |  | 12.8 | Describe any sensitivity analyses. |
|  |  | 12.9 | If relevant, report methods used to account for adjustment for assay performance (sensitivity and specificity), the probability of seropositivity or seroconversion if infected, and to account for decay in antibody titers over time. |
| Domain 3: Laboratory methods | Sample type and handling | 13.1 | Describe the sample type—whole blood, dried blood, serum or plasma. If plasma is used, specify the anticoagulant used (heparin, sodium citrate, EDTA, etc). |
|  |  | 13.2 | Describe the specimen storage conditions (4°C, −20°C, −80°C). If frozen prior to the analysis, describe the time to freezing and the number of freeze/thaw cycles prior to testing. |
|  | Serological assays | 13.3 | Wherever possible, use defined and standardized methods that have been established in more than one laboratory, and that ideally are commercially available in more than one country. Avoid laboratory‐level formulations if standardized formulations are available for the same analytical targets. |
|  |  | 13.4 | Specify the testing algorithm (if more than one test used) and assay type (eg, virus neutralization/microneutralization/surrogate neutralization; ELISA; LFIA; CLIA; other) and readout used to determine the endpoint titer. |
|  |  | 13.5 | Reference a previously published protocol, if used, and any modifications of the protocol. If a previously published protocol was not used, provide full details in supplementary materials. For in‐house assays, include a description of the assay format (e.g., direct or indirect immunoassay) as well as description of cutoff determination and which antibody isotype is targeted, and reference previously published validation data. |
|  |  | 13.6 | State what is known about the determinants of the variability of the antibody detection assay being used. |
|  |  | 13.7 | Specify the antigen(s) and antibody isotope target used, with standardized nomenclature and reference; specify whether live virus or pseudo virus was used (where applicable). Describe how the cutoff was established. If viral antigen produced in‐house is used, specify sequence, expression system (bacteria or mammalian cells). Specify reactivity with other coronavirus antigens (MERS‐CoV, SARS‐CoV, seasonal CoVs) in the same population. |
|  |  | 13.8 | Describe positive and negative controls used. Specify international standards used, if appropriate. |
|  |  | 13.9 | Describe starting and end dilutions. |
|  |  | 13.10 | Specify laboratory biosafety conditions. |
|  |  | 13.11 | Specify whether replication was performed, and if so, the acceptable replication parameters. |
|  |  | 13.12 | Specify whether a confirmatory assay was performed and all specifics of this assay, at the same level of detail. |
| Domain 4: Results | Participants | 13.13 | Report the numbers of individuals at each stage of the study—the numbers potentially eligible, examined for eligibility, confirmed eligible, included in the study, completing follow‐up, and analyzed. |
|  |  | 13.14 | Give reasons for non‐participation at each stage. Consider use of a flow diagram |
|  | Descriptive data | 14.1 | Give characteristics of study participants (eg, demographic, clinical, social) and information on exposures and potential risk factors for all participants, not solely stratified by outcome status. |
|  |  | 14.2 | Indicate the number of participants with missing data for each variable of interest. |
|  |  | 14.3 | For a cohort study, detail follow‐up time (eg, average and total amount). |
|  | Outcome data | 15.1 | For a cohort study, report the numbers of outcome events or summary measures over time. |
|  |  | 15.2 | For a case‐control study, report the numbers in each exposure category, or summary measures of exposure. |
|  |  | 15.3 | For a cross‐sectional study, report the numbers of outcome events or summary measures. |
|  | Main result | 16.1 | Report unadjusted estimates of distribution of seropositivity by age group. |
|  |  | 16.2 | Report methods to standardize the results from the study sample to the target population. |
|  | Other analyses | 17.1 | Report other analyses performed—analyses of subgroups and interactions, and sensitivity analyses. |
| Domain 5: Discussion | Key results | 18.1 | Summarize key results with reference to study objectives. |
|  | Limitations | 19.1 | Discuss limitations and strengths of the study. |
|  | Interpretation | 20.1 | Discuss the interpretation of the results in the context of known or potential cross‐reactivity, assay performance and other sources of bias |
|  | Generalizability | 21.1 | Discuss the generalizability (external validity) of the study results. |
| Domain 6: Other information | Ethics approval | 22.1 | Specify if institutional review board approval was received; if not, specify reason (eg, public health outbreak response/non‐research designation). |

# **Supplementary file 2: Beta regression methods**

Beta regression was performed using the betareg package. Univariable models were constructed for all candidate predictors. Among them, publication date was used in a continuous piecewise model with two knots, one at the date of ROSES-S publication and the other 154 days later, to assess trends of adherence to ROSES-S over time. This model was compared to a non-segmented model using the likelihood-ratio test. The same analysis was repeated with a publication time lag of 255 days for its second knot as a sensitivity analysis. Publication date was entered in these models as a date variable and its coefficients were multiplied by 30.4 to represent monthly increments.

A two-step strategy was used to fit the mean and the precision beta regression sub-models. The mean component was fit first, assuming constant dispersion. The MuMIN package was used for a best subset model building approach with AIC as the selection criterion. When multiple models were within two delta AIC of the top model, the most parsimonious one was chosen. Then, the finalized mean sub-model was held constant while selecting the regressors for the precision sub-model using the same best subset approach. Finally, marginal effects with 95% confidence intervals were calculated using the mfx package to facilitate the interpretation of average change in total adherence scores at percentage level.

#

# **Supplementary file 3: Reference list of included studies**

1. Sydney ER, Kishore P, Laniado I, Rucker LM, Bajaj K, Zinaman MJ. Antibody evidence of SARS-CoV-2 infection in healthcare workers in the Bronx. *Infection Control & Hospital Epidemiology*. 2020;41(11):1348-1349. doi:[10.1017/ice.2020.437](https://doi.org/10.1017/ice.2020.437).

2. Canadian Blood Services CBS. *COVID-19 Seroprevalence Report 1– August 19, 2020*.; 2020. <https://www.covid19immunitytaskforce.ca/wp-content/uploads/2020/09/COVID-19-Public-Report_2.pdf>.

3. Loyola EMV, Cruz IM, Cabrejos VC, Pacheco HM, Benitez MA, Chomba MP. Gestación en tiempos de pandemia COVID-19. Hospital Nacional Docente Madre Niño San Bartolomé, Lima, Perú. *Revista Peruana de Ginecología y Obstetricia*. 2020;66(3). doi:[10.31403/rpgo.v66i2265](https://doi.org/10.31403/rpgo.v66i2265).

4. Brutto OHD, Costa AF, Mera RM, Recalde BY, Bustos JA, García HH. Late incidence of SARS-CoV-2 infection in a highly-endemic remote rural village. A prospective population-based cohort study. *Pathogens and Global Health*. 2020;114(8):457-462. doi:[10.1080/20477724.2020.1826152](https://doi.org/10.1080/20477724.2020.1826152).

5. Stock AD, Bader ER, Cezayirli P, et al. COVID-19 Infection Among Healthcare Workers: Serological Findings Supporting Routine Testing. *Frontiers in Medicine*. 2020;7. doi:[10.3389/fmed.2020.00471](https://doi.org/10.3389/fmed.2020.00471).

6. Lackermair K, William F, Grzanna N, et al. Infection with SARS-CoV-2 in primary care health care workers assessed by antibody testing. *Family Practice*. August 2020:cmaa078. doi:[10.1093/fampra/cmaa078](https://doi.org/10.1093/fampra/cmaa078).

7. Nsn G, C J, R M, et al. High rates of SARS-CoV-2 seropositivity in nursing home residents. *Journal of Infection*. August 2020:S0163445320305740. doi:[10.1016/j.jinf.2020.08.040](https://doi.org/10.1016/j.jinf.2020.08.040).

8. Statistica IN di. *PRIMI RISULTATI DELL’INDAGINE DI SIEROPREVALENZA SUL SARS-CoV-2*. Instituto Nazionale di Statistica; 2020. <https://www.istat.it/it/files//2020/08/ReportPrimiRisultatiIndagineSiero.pdf>. Accessed May 13, 2021.

9. Carozzi FM, Cusi MG, Pistello M, et al. Detection of asymptomatic SARS-CoV-2 infections among healthcare workers: Results from a large-scale screening program based on rapid serological testing. *medRxiv*. August 2020. doi:[10.1101/2020.07.30.20149567](https://doi.org/10.1101/2020.07.30.20149567).

10. Reiter T, Pajenda S, Wagner L, et al. Covid-19 serology in nephrology health care workers. *medRxiv*. July 2020:2020.07.21.20136218. doi:[10.1101/2020.07.21.20136218](https://doi.org/10.1101/2020.07.21.20136218).

11. Agency SPH. *Påvisning Av Antikroppar Efter Genomgången Covid-19 Hos Blodgivare (Delrapport 1)*. Swedish Public Health Agency; 2020. <https://www.folkhalsomyndigheten.se/contentassets/9c5893f84bd049e691562b9eeb0ca280/pavisning-antikroppar-genomgangen-covid-19-blodprov-oppenvarden-delrapport-1.pdf>.

12. Public Health England. National COVID-19 surveillance report: 9 July 2020 (week 28). July 2020. <https://www.gov.uk/government/publications/national-covid-19-surveillance-reports>.

13. Moncunill G, Mayor A, Santano R, et al. SARS-CoV-2 infections and antibody responses among health care workers in a Spanish hospital after a month of follow-up. *medRxiv*. August 2020:2020.08.23.20180125. doi:[10.1101/2020.08.23.20180125](https://doi.org/10.1101/2020.08.23.20180125).

14. Tsaneva-Damyanova D. SARS-CoV-2: Seroepidemiological pattern in northeastern Bulgaria. *Biotechnology & Biotechnological Equipment*. 2020;34(1):441-446. doi:[10.1080/13102818.2020.1772105](https://doi.org/10.1080/13102818.2020.1772105).

15. Younas A, Waheed S, Khawaja S, Imam M, Borhany M, Shamsi T. Seroprevalence of SARS-CoV-2 antibodies among healthy blood donors in Karachi, Pakistan. *Transfusion and Apheresis Science*. 2020;59(6):102923. doi:[10.1016/j.transci.2020.102923](https://doi.org/10.1016/j.transci.2020.102923).

16. Petersen MS, Strøm M, Christiansen DH, et al. Seroprevalence of SARS-CoV-2–Specific Antibodies, Faroe Islands. *Emerging Infectious Diseases*. 2020;26(11):2760-2762. doi:[10.3201/eid2611.202736](https://doi.org/10.3201/eid2611.202736).

17. Kern PM, Müller H-H, Menzel T, Weisser H. Studie zur Immunität gegen SARS-CoV-2: Keine signifikante humorale Immunität gegen SARS-CoV-2 im medizinischen Personal eines Klinikums der Maximalversorgung und in der Stadtregion Fulda. *Der Klinikarzt*. 2020;49(06):268-273. doi:[10.1055/a-1198-1243](https://doi.org/10.1055/a-1198-1243).

18. Matsuba I, Hatori N, Koido N, et al. Survey of the current status of subclinical coronavirus disease 2019 (COVID-19). *Journal of Infection and Chemotherapy*. 2020;26(12):1294-1300. doi:[10.1016/j.jiac.2020.09.005](https://doi.org/10.1016/j.jiac.2020.09.005).

19. Gonçalves J, Sousa RL, Jacinto MJ, et al. Evaluating SARS-CoV-2 Seroconversion Following Relieve of Confinement Measures. *Frontiers in Medicine*. 2020;7. doi:[10.3389/fmed.2020.603996](https://doi.org/10.3389/fmed.2020.603996).

20. Isgrò MA, Vitale MG, Celentano E, et al. Immunotherapy may protect cancer patients from SARS-CoV-2 infection: A single-center retrospective analysis. *Journal of Translational Medicine*. 2021;19(1):132. doi:[10.1186/s12967-021-02798-2](https://doi.org/10.1186/s12967-021-02798-2).

21. Häusler S, Weigl M, Ambrosch A, Gruber R, Seelbach-Göbel B, Malfertheiner SF. Peripartal anti-SARS-CoV-2-IgA/IgG in asymptomatic pregnant women during regional SARS-CoV-2-outbreak. *Journal of Perinatal Medicine*. February 2021. doi:[10.1515/jpm-2021-0001](https://doi.org/10.1515/jpm-2021-0001).

22. Sulcebe G, Ylli A, Cenko F, Kurti-Prifti M. Rapid increase of SARS-CoV-2 seroprevalence during the 2020 pandemic year in the population of the city of Tirana, Albania. *medRxiv*. February 2021. doi:[10.1101/2021.02.18.21251776](https://doi.org/10.1101/2021.02.18.21251776).

23. Craigie A, McGregor R, Whitcombe A, et al. SARS-CoV-2 antibodies in the Southern Region of New Zealand, 2020. *medRxiv*. January 2020:2020.10.20.20215616. doi:[10.1101/2020.10.20.20215616](https://doi.org/10.1101/2020.10.20.20215616).

24. Trieu M-C, Bansal A, Madsen A, et al. SARS-CoV-2–Specific Neutralizing Antibody Responses in Norwegian Health Care Workers After the First Wave of COVID-19 Pandemic: A Prospective Cohort Study. *The Journal of Infectious Diseases*. 2020;2021-(jiaa737). doi:[10.1093/infdis/jiaa737](https://doi.org/10.1093/infdis/jiaa737).

25. Turner-Stokes T, Jiang E, Johnson N, et al. Serologic Screening for Coronavirus Disease 2019 in Patients With Glomerular Disease. *Kidney International Reports*. 2021;6(5):1402-1406. doi:[10.1016/j.ekir.2021.02.006](https://doi.org/10.1016/j.ekir.2021.02.006).

26. Varona JF, Madurga R, Peñalver F, et al. Seroprevalence of SARS-CoV-2 antibodies in over 6000 healthcare workers in Spain. *International Journal of Epidemiology*. 2021;50(2):400-409. doi:[10.1093/ije/dyaa277](https://doi.org/10.1093/ije/dyaa277).

27. Caramelli B, Escalante-Rojas MC, Chauhan HKC, Siciliano RF, Bittencourt MS, Micelli AC. The "false-positive" conundrum: IgA reference level overestimates the seroprevalence of antibodies to SARS-CoV-2. *Journal of Global Health*. 2021;11:05001. doi:[10.7189/jogh.11.05001](https://doi.org/10.7189/jogh.11.05001).

28. Mullins KE, Merrill V, Ward M, et al. Validation of COVID-19 serologic tests and large scale screening of asymptomatic healthcare workers. *Clinical Biochemistry*. 2021;90:23-27. doi:[10.1016/j.clinbiochem.2021.01.004](https://doi.org/10.1016/j.clinbiochem.2021.01.004).

29. Region TS. *6 April*. The Stockholm Region; 2021. <https://www.regionstockholm.se/verksamhet/halsa-och-vard/nyheter-lagesrapporter-covid-19/2021/04/6-april-lagesrapport-om-covid-19/>. Accessed November 22, 2021.

30. Region TS. *27 April*. The Stockholm Region; 2021. <https://www.regionstockholm.se/verksamhet/halsa-och-vard/nyheter-lagesrapporter-covid-19/2021/04/27-april-Lagesrapport-om-ovid-19/>. Accessed November 23, 2021.

31. Xu L, Doyle J, Barbeau DJ, et al. A Cross-Sectional Study of SARS-CoV-2 Seroprevalence between Fall 2020 and February 2021 in Allegheny County, Western Pennsylvania, USA. *Pathogens*. 2021;10(6):710. doi:[10.3390/pathogens10060710](https://doi.org/10.3390/pathogens10060710).

32. Sendi P, Baldan R, Thierstein M, et al. A Multidimensional Cross-Sectional Analysis of Coronavirus Disease 2019 Seroprevalence Among a Police Officer Cohort: The PoliCOV-19 Study. *Open Forum Infectious Diseases*. 2021;8(12):ofab524. doi:[10.1093/ofid/ofab524](https://doi.org/10.1093/ofid/ofab524).

33. Hippich M, Sifft P, Zapardiel-Gonzalo J, et al. A public health antibody screening indicates a marked increase of SARS-CoV-2 exposure rate in children during the second wave. *Med*. 2021;2(5):571-572. doi:[10.1016/j.medj.2021.03.019](https://doi.org/10.1016/j.medj.2021.03.019).

34. Miraglia JL, Nascimento Monteiro C, Giannecchini Romagnolo A, et al. A seroprevalence survey of anti-SARS-CoV-2 antibodies among individuals 18 years of age or older living in a vulnerable region of the city of São Paulo, Brazil. *PLOS ONE*. 2021;16(7):1-9. doi:[10.1371/journal.pone.0255412](https://doi.org/10.1371/journal.pone.0255412).

35. Boey L, Roelants M, Merckx J, et al. Age-dependent seroprevalence of SARS-CoV-2 antibodies in school-aged children from areas with low and high community transmission. *European Journal of Pediatrics*. 2022;181(2):571-578. doi:[10.1007/s00431-021-04222-9](https://doi.org/10.1007/s00431-021-04222-9).

36. Nasrallah GK, Dargham SR, Shurrab F, et al. Analytic comparison between three high-throughput commercial SARS-CoV-2 antibody assays reveals minor discrepancies in a high-incidence population. *Scientific Reports*. 2021;11(1):11837. doi:[10.1038/s41598-021-91235-x](https://doi.org/10.1038/s41598-021-91235-x).

37. Alenda R, Gonzalez-Diez R, Richart A, et al. P-216 Anti SARS-CoV-2 antibodies prevalence in Madrid blood donors prior to first outbreak. *Vox Sanguinis*. 2021;116(S1):5-188. <https://onlinelibrary-wiley-com.proxy3.library.mcgill.ca/doi/10.1111/vox.13117>. Accessed June 29, 2021.

38. Guzijan G, Marisavljević D, Milosavić M, Jovanović-Srzentić S. Anti-SARS-COV-2 IgG seroprevalence study among blood donors in the Republic of Srpska: A 30 days survey. *Vojnosanitetski pregled*. 2021;78(6):691-692. doi:[10.2298/VSP200122059G](https://doi.org/10.2298/VSP200122059G).

39. Soeorg H, Jõgi P, Naaber P, Ottas A, Toompere K, Lutsar I. Seroprevalence and levels of IgG antibodies after COVID-19 infection or vaccination. *Infectious Diseases*. 2022;54(1):63-71. doi:[10.1080/23744235.2021.1974540](https://doi.org/10.1080/23744235.2021.1974540).

40. Mueller T. Antibodies against severe acute respiratory syndrome coronavirus type 2 (SARS-CoV-2) in individuals with and without COVID-19 vaccination: A method comparison of two different commercially available serological assays from the same manufacturer. *Clinica Chimica Acta*. 2021;518:9-16. doi:[10.1016/j.cca.2021.03.007](https://doi.org/10.1016/j.cca.2021.03.007).

41. Pérez-García F, Pérez-Zapata A, Arcos Varela N, et al. [[SARS-CoV-2 seroprevalence among workers in a hospital in Madrid.]](https://www.ncbi.nlm.nih.gov/pubmed/34675168). *Revista Espanola De Salud Publica*. 2021;95:e202110176.

42. Barron D, Richards O, Archer F, Abdelrazek M, Ranjan R, Omolokun O. A cluster of children with facial nerve palsy in a high prevalence area for COVID-19. *BMC Pediatrics*. 2021;21(1):470. doi:[10.1186/s12887-021-02831-9](https://doi.org/10.1186/s12887-021-02831-9).

43. Fisher M, Levy H, Fatelevich E, et al. A Serological Snapshot of COVID-19 Initial Stages in Israel by a 6-Plex Antigen Array. *Microbiology Spectrum*. 2021;0(0):e00870-21. doi:[10.1128/Spectrum.00870-21](https://doi.org/10.1128/Spectrum.00870-21).

44. Montague BT, Wipperman MF, Hooper AT, et al. Anti-SARS-CoV-2 IgA Identifies Asymptomatic Infection in First Responders. *The Journal of Infectious Diseases*. 2021;(jiab524). doi:[10.1093/infdis/jiab524](https://doi.org/10.1093/infdis/jiab524).

45. Kugeler KJ, Podewils LJ, Alden NB, et al. Assessment of SARS-CoV-2 Seroprevalence by Community Survey and Residual Specimens, Denver, Colorado, July–August 2020. *Public Health Reports*. 2022;137(1):128-136. doi:[10.1177/00333549211055137](https://doi.org/10.1177/00333549211055137).

46. Fryatt A, Simms V, Bandason T, et al. Community SARS-CoV-2 seroprevalence before and after the second wave of SARS-CoV-2 infection in Harare, Zimbabwe. *EClinicalMedicine*. 2021;41:101172. doi:[10.1016/j.eclinm.2021.101172](https://doi.org/10.1016/j.eclinm.2021.101172).

47. Velay A, Gallais F, Wendling M, et al. COVID-19 exposure in SARS-CoV-2-seropositive hospital staff members during the first pandemic wave at Strasbourg University Hospital, France. *Infectious Diseases Now*. November 2021. doi:[10.1016/j.idnow.2021.11.002](https://doi.org/10.1016/j.idnow.2021.11.002).

48. Durand GA, Laval F de, Bonet d’Oléon A de, et al. COVID-19 outbreak among French firefighters, Marseille, France, 2020. *Eurosurveillance*. 2021;26(41). doi:[10.2807/1560-7917.ES.2021.26.41.2001676](https://doi.org/10.2807/1560-7917.ES.2021.26.41.2001676).

49. Canadian Blood Services. *COVID-19 Seroprevalence Report October 13, 2021 Report #12 - July 2021 Survey Tracking Seroprevalence in the Vaccine Era*.; 2021.

50. Stead D, Adeniyi OV, Singata-Madliki M, et al. *Cumulative Incidence of SARS-CoV-2 and Associated Risk Factors Among Healthcare Workers in the Eastern Cape, South Africa*.; 2021:2021.11.08.21265966. doi:[10.1101/2021.11.08.21265966](https://doi.org/10.1101/2021.11.08.21265966).

51. Pagani G, Conti F, Giacomelli A, et al. Differences in the Prevalence of SARS-CoV-2 Infection and Access to Care between Italians and Non-Italians in a Social-Housing Neighbourhood of Milan, Italy. *International Journal of Environmental Research and Public Health*. 2021;18(20):10621. doi:[10.3390/ijerph182010621](https://doi.org/10.3390/ijerph182010621).

52. Dehnen D, Dehnen K, Trilling M, et al. Discrepancy between frequent occurrence of COVID‐19‐like symptoms and low seroconversion rates among healthcare workers. *Journal of Medical Virology*. October 2021:jmv.27385. doi:[10.1002/jmv.27385](https://doi.org/10.1002/jmv.27385).

53. Elfström KM, Blomqvist J, Nilsson P, et al. Differences in risk for SARS-CoV-2 infection among healthcare workers. *medRxiv*. April 2021. doi:[10.1101/2021.03.30.21254653](https://doi.org/10.1101/2021.03.30.21254653).

54. Gallian P, Pastorino B, Morel P, Chiaroni J, Ninove L, Lamballerie X de. Lower prevalence of antibodies neutralizing SARS-CoV-2 in group O French blood donors. *Antiviral Research*. 2020;181:104880. doi:[10.1016/j.antiviral.2020.104880](https://doi.org/10.1016/j.antiviral.2020.104880).

55. Reifer J, Hayum N, Heszkel B, Klagsbald I, Streva VA. SARS-CoV-2 IgG antibody responses in New York City. *Diagnostic Microbiology and Infectious Disease*. 2020;98(3):115128. doi:[10.1016/j.diagmicrobio.2020.115128](https://doi.org/10.1016/j.diagmicrobio.2020.115128).

56. Khalil A, Hill R, Wright A, Ladhani S, O’Brien P. SARS-CoV-2-Specific Antibody Detection in Healthcare Workers in a UK Maternity Hospital: Correlation With SARS-CoV-2 RT-PCR Results. *Clinical Infectious Diseases*. 2020;(ciaa893). doi:[10.1093/cid/ciaa893](https://doi.org/10.1093/cid/ciaa893).

57. Dixit JV, Iravane J, Andurkar SP, et al. Seroprevalence of COVID-19 in Aurangabad District. *International Journal of Creative Research Thoughts (IJCRT)*. 2020;8(9):3675-3683. <https://ijcrt.org/papers/IJCRT2009467.pdf>.

58. Bahrs C, Kimmig A, Weis S, et al. Seroprevalence of SARS CoV-2 antibodies in healthcare workers and administration employees: A prospective surveillance study at a 1,400-bed university hospital in Germany. *medRxiv*. September 2020. doi:[10.1101/2020.09.29.20203737](https://doi.org/10.1101/2020.09.29.20203737).

59. Dingens AS, Crawford KHD, Adler A, et al. Seroprevalence of SARS-CoV-2 among children visiting a hospital during the initial Seattle outbreak. *medRxiv*. May 2020. doi:[10.1101/2020.05.26.20114124](https://doi.org/10.1101/2020.05.26.20114124).

60. Silva AP, Fernanda Aguirre M, Ballejo C, et al. Seroprevalence of the SARS-CoV-2 infection in health workers of the Sanitary Region VIII, at province of Buenos Aires. *medRxiv*. January 2020:2020.09.07.20189050. doi:[10.1101/2020.09.07.20189050](https://doi.org/10.1101/2020.09.07.20189050).

61. Zhou F, Li J, Lu M, et al. Tracing asymptomatic SARS-CoV-2 carriers among 3674 hospital staff:a cross-sectional survey. *EClinicalMedicine*. 2020;26. doi:[10.1016/j.eclinm.2020.100510](https://doi.org/10.1016/j.eclinm.2020.100510).

62. Aatresh AV, Cummings K, Gerstein H, et al. Development of at-home sample collection logistics for large scale SARS-CoV-2 seroprevalence studies. *medRxiv*. January 2021. doi:[10.1101/2021.01.14.21249824](https://doi.org/10.1101/2021.01.14.21249824).

63. Cremoni M, Ruetsch C, Zorzi K, et al. Humoral and Cellular Response of Frontline Health Care Workers Infected by SARS-CoV-2 in Nice, France: A Prospective Single-Center Cohort Study. *Frontiers in Medicine*. 2021;7. doi:[10.3389/fmed.2020.608804](https://doi.org/10.3389/fmed.2020.608804).

64. Choi M, Bachmann F, Naik MG, et al. Low Seroprevalence of SARS-CoV-2 Antibodies during Systematic Antibody Screening and Serum Responses in Patients after COVID-19 in a German Transplant Center. *Journal of Clinical Medicine*. 2020;9(11):3401. doi:[10.3390/jcm9113401](https://doi.org/10.3390/jcm9113401).

65. Ripperger TJ, Uhrlaub JL, Watanabe M, et al. Orthogonal SARS-CoV-2 Serological Assays Enable Surveillance of Low-Prevalence Communities and Reveal Durable Humoral Immunity. *Immunity*. 2020;53(5):925-933.e4. doi:[10.1016/j.immuni.2020.10.004](https://doi.org/10.1016/j.immuni.2020.10.004).

66. Tönshoff B, Müller B, Elling R, et al. Prevalence of SARS-CoV-2 Infection in Children and Their Parents in Southwest Germany. *JAMA Pediatrics*. 2021;175(6):586-593. doi:[10.1001/jamapediatrics.2021.0001](https://doi.org/10.1001/jamapediatrics.2021.0001).

67. Wong S-Y, Gold S, Accorsi EK, et al. Safety of administering biologics to IBD patients at an outpatient infusion center In New York City during the COVID-19 pandemic: Sars-CoV-2 seroprevalence and clinical and social characteristics. *medRxiv*. March 2021:2021.03.15.21253615. doi:[10.1101/2021.03.15.21253615](https://doi.org/10.1101/2021.03.15.21253615).

68. Ralli M, Arcangeli A, Soave PM, Voglino MC, De-Giorgio F. SARS-CoV-2 seroprevalence in the Vatican City State. *European Journal of Internal Medicine*. 2021;0(0). doi:[10.1016/j.ejim.2021.01.029](https://doi.org/10.1016/j.ejim.2021.01.029).

69. Santena F, Lopes J, Perez M, et al. Seroconversion for SARS-CoV-2 in rheumatic patients on synthetic and biologics Disease Modifying Anti-Rheumatic Drugs in São Paulo, Brazil. October 2020. doi:[10.21203/rs.3.rs-97191/v1](https://doi.org/10.21203/rs.3.rs-97191/v1).

70. Ladoire S, Goussot V, Redersdorff E, et al. Seroprevalence of SARS-CoV-2 among the staff and patients of a French cancer centre after first lockdown: The canSEROcov study. *European Journal of Cancer*. 2021;148:359-370. doi:[10.1016/j.ejca.2021.02.027](https://doi.org/10.1016/j.ejca.2021.02.027).

71. El Bouzidi K, Pirani T, Rosadas C, et al. Severe Acute Respiratory Syndrome Coronavirus-2 Infections in Critical Care Staff: Beware the Risks Beyond the Bedside. *Critical Care Medicine*. 2021;49(3):428-436. doi:[10.1097/CCM.0000000000004878](https://doi.org/10.1097/CCM.0000000000004878).

72. Buss LF, Prete CA, Abrahim CMM, et al. Three-quarters attack rate of SARS-CoV-2 in the Brazilian Amazon during a largely unmitigated epidemic. *Science*. 2021;371(6526):288-292. doi:[10.1126/science.abe9728](https://doi.org/10.1126/science.abe9728).

73. Moyet J, Joseph C, Brochot E, et al. Sérologie Covid-19 en Ehpad et soins de longue durée : Prévalence de la séroconversion au CHU Amiens-Picardie. *Gériatrie et Psychologie Neuropsychiatrie du Vieillissement*. 2016;1(1). doi:[10.1684/pnv.2021.0952](https://doi.org/10.1684/pnv.2021.0952).

74. Region TS. *20 April*. The Stockholm Region; 2021. <https://www.regionstockholm.se/verksamhet/halsa-och-vard/nyheter-lagesrapporter-covid-19/2021/04/mall-lagesrapport-med-tabell/>. Accessed November 23, 2021.

75. Kianersi S, Ludema C, Macy JT, et al. A Cross-Sectional Analysis of Demographic and Behavioral Risk Factors of Severe Acute Respiratory Syndrome Coronavirus 2 Seropositivity Among a Sample of U.S. College Students. *Journal of Adolescent Health*. 2021;69(2):219-226. doi:[10.1016/j.jadohealth.2021.05.003](https://doi.org/10.1016/j.jadohealth.2021.05.003).

76. Labriola L, Scohy A, Seghers F, et al. A Longitudinal, 3-Month Serologic Assessment of SARS-CoV-2 Infections in a Belgian Hemodialysis Facility. *Clinical Journal of the American Society of Nephrology*. 2021;16(4):613-614. doi:[10.2215/CJN.12490720](https://doi.org/10.2215/CJN.12490720).

77. Brondino N, Bertoglio F, Forneris F, et al. A Pilot Study on Covid and Autism: Prevalence, Clinical Presentation and Vaccine Side Effects. *Brain Sciences*. 2021;11(7):860. doi:[10.3390/brainsci11070860](https://doi.org/10.3390/brainsci11070860).

78. Li M-C, Lee N-Y, Tsai W-L, Ko W-C. A seroprevalence study of COVID-19 at a campus in southern Taiwan. *Journal of Microbiology, Immunology, and Infection*. 2021;54(5):1008-1010. doi:[10.1016/j.jmii.2021.03.018](https://doi.org/10.1016/j.jmii.2021.03.018).

79. Chanchlani N, Lin S, Chee D, et al. Adalimumab and infliximab impair SARS-CoV-2 antibody responses: Results from a therapeutic drug monitoring study in 11422 biologic-treated patients. *Journal of Crohn’s and Colitis*. September 2021. doi:[10.1093/ecco-jcc/jjab153](https://doi.org/10.1093/ecco-jcc/jjab153).

80. Stephan Gehring, Omar Okasha, Frank Kowalzik, et al. An Epidemiological Cohort Study of SARS-CoV-2 and COVID-19 in German Healthcare Workers – Interim Analysis after Six Months of Follow-up. *Scientific Reports*. August 2021. doi:[10.21203/rs.3.rs-688656/v1](https://doi.org/10.21203/rs.3.rs-688656/v1).

81. Aryal S, Pandit S, Pokhrel S, et al. ANTI - SARS-CoV-2 ANTIBODY SCREENING IN HEALTH CARE WORKERS AND ITS CORRELATION WITH CLINICAL PRESENTATION. July 2021. doi:[10.1101/2021.07.01.21259884](https://doi.org/10.1101/2021.07.01.21259884).

82. Messaaoui A, Hajselova L, Tenoutasse S. Anti-SARS-CoV-2 antibodies in new-onset type 1 diabetes in children during pandemic in Belgium. *Journal of Pediatric Endocrinology and Metabolism*. 2021;0(0):000010151520210289. doi:[10.1515/jpem-2021-0289](https://doi.org/10.1515/jpem-2021-0289).

83. Kagucia EW, Gitonga JN, Kalu C, et al. Anti-Severe Acute Respiratory Syndrome Coronavirus 2 Immunoglobulin G Antibody Seroprevalence Among Truck Drivers and Assistants in Kenya. *Open Forum Infectious Diseases*. 2021;8(7). doi:[10.1093/ofid/ofab314](https://doi.org/10.1093/ofid/ofab314).

84. Brune B, Korth J, Fessmann K, et al. SARS-CoV-2-IgG-Antikörperseroprävalenz bei Personal in der außerklinischen Bekämpfung der COVID-19-Pandemie. *Notfall + Rettungsmedizin*. October 2021. doi:[10.1007/s10049-021-00948-z](https://doi.org/10.1007/s10049-021-00948-z).

85. Harboun M, Verdun S, Brénière V, Luquel L, Jourdan M, De Malherbe A. Séroprévalence, facteurs de risque et présentation clinique après la première vague COVID-19 dans les EHPAD du groupe UNIVI : Étude SEROCOVID. *La Revue de Médecine Interne*. October 2021:S0248866321010547. doi:[10.1016/j.revmed.2021.10.330](https://doi.org/10.1016/j.revmed.2021.10.330).

86. Wattal C, Oberoi JK, Goel N, et al. A cross-sectional study of SARS-CoV-2 seroprevalence among asymptomatic healthcare workers in a tertiary healthcare centre: Assessing the impact of PPE guidelines. *Indian Journal of Medical Microbiology*. October 2021. doi:[10.1016/j.ijmmb.2021.09.011](https://doi.org/10.1016/j.ijmmb.2021.09.011).

87. Chandrasingh S, George CE, Inbaraj LR. Are hospitals epicentres of COVID19 transmission? Findings of serial serosurveys among healthcare workers from a tertiary hospital in South India. *Indian Journal of Medical Microbiology*. November 2021. doi:[10.1016/j.ijmmb.2021.10.006](https://doi.org/10.1016/j.ijmmb.2021.10.006).

88. Kogevinas M, Casta ño-VG, Karachaliou M, et al. Ambient Air Pollution in Relation to SARS-CoV-2 Infection, Antibody Response, and COVID-19 Disease: A Cohort Study in Catalonia, Spain (COVICAT Study). *Environmental Health Perspectives*. 2021;129(11):117003. doi:[10.1289/EHP9726](https://doi.org/10.1289/EHP9726).

89. Sam I-C, Chong YM, Abdullah A, et al. Changing predominant SARS-CoV-2 lineages drives successive COVID-19 waves in Malaysia, February 2020 to March 2021. *Journal of Medical Virology*. 2021;94(3):1146-1153. doi:[10.1002/jmv.27441](https://doi.org/10.1002/jmv.27441).

90. Hoffmann S, Schiebel J, Hufert F, Gremmels H-D, Spallek J. COVID-19 among Healthcare Workers: A Prospective Serological-Epidemiological Cohort Study in a Standard Care Hospital in Rural Germany. *International Journal of Environmental Research and Public Health*. 2021;18(20):10999. doi:[10.3390/ijerph182010999](https://doi.org/10.3390/ijerph182010999).

91. Bansal N, Ovchinsky N, Foca M, et al. COVID-19 infection in pediatric solid organ transplant patients. *Pediatric Transplantation*. 2021;n/a(n/a):e14156. doi:[10.1111/petr.14156](https://doi.org/10.1111/petr.14156).

92. Nopsopon T, Pongpirul K, Chotirosniramit K, Hiransuthikul N. COVID-19 seroprevalence among hospital staff and preprocedural patients in Thai community hospitals: A cross-sectional study. *BMJ Open*. 2021;11(10):e046676. doi:[10.1136/bmjopen-2020-046676](https://doi.org/10.1136/bmjopen-2020-046676).

93. Bryan A, Tatem K, Diuguid-Gerber J, et al. Cross-sectional study evaluating the seroprevalence of SARS-CoV-2 antibodies among healthcare workers and factors associated with exposure during the first wave of the COVID-19 pandemic in New York. *BMJ Open*. 2021;11(11):e053158. doi:[10.1136/bmjopen-2021-053158](https://doi.org/10.1136/bmjopen-2021-053158).

94. Zöllkau J, Baier M, Scherag A, Schleußner E, Groten T. Periodenprävalenz von SARS-CoV-2 in einer unselektierten Stichprobe schwangerer Frauen in Jena, Thüringen. *Zeitschrift für Geburtshilfe und Neonatologie*. 2020;224(04):194-198. doi:[10.1055/a-1206-1033](https://doi.org/10.1055/a-1206-1033).

95. Public Health England. National COVID-19 surveillance report: 11 September 2020 (week 37). September 2020. <https://www.gov.uk/government/publications/national-covid-19-surveillance-reports>.

96. Hsiao M, Davies M-A, Kalk E, et al. SARS-CoV-2 seroprevalence in the Cape Town Metropolitan sub-districts after the peak of infections. *NICD COVID-19 Special Public Health Surveillance Bulletin*. 2020;18(Supplementary issue 5):1-9. <https://www.nicd.ac.za/wp-content/uploads/2020/09/COVID-19-Special-Public-Health-Surveillance-Bulletin_Issue-5.pdf>.

97. Zurita C, Guarderas P, Sevillano G, Ortega-Paredes D, Zurita J. SEROPREVALENCE OF ANTI-SARS-COV-2 IGG ANTIBODIES IN ECUADORIAN POPULATION. 2020. doi:[10.13140/RG.2.2.12356.24968](https://doi.org/10.13140/RG.2.2.12356.24968).

98. Mesnil M, Joubel K, Yavchitz A, Miklaszewski N, Devys J-M. Seroprevalence of SARS-Cov-2 in 646 professionals at the Rothschild Foundation Hospital (ProSeCoV study). *Anaesthesia Critical Care & Pain Medicine*. 2020;39(5):595-596. doi:[10.1016/j.accpm.2020.08.003](https://doi.org/10.1016/j.accpm.2020.08.003).

99. Mahajan S, Srinivasan R, Redlich CA, et al. Seroprevalence of SARS-CoV-2-Specific IgG Antibodies Among Adults Living in Connecticut Between March 1 and June 1, 2020: Post-Infection Prevalence (PIP) Study. *medRxiv*. August 2020:2020.08.04.20168203. doi:[10.1101/2020.08.04.20168203](https://doi.org/10.1101/2020.08.04.20168203).

100. Dalla Volta A, Valcamonico F, Pedersini R, et al. The Spread of SARS-CoV-2 Infection Among the Medical Oncology Staff of ASST Spedali Civili of Brescia: Efficacy of Preventive Measures. *Frontiers in Oncology*. 2020;10:1574. doi:[10.3389/fonc.2020.01574](https://doi.org/10.3389/fonc.2020.01574).

101. Schaffner A, Risch L, Aeschbacher S, et al. Characterization of a Pan-Immunoglobulin Assay Quantifying Antibodies Directed against the Receptor Binding Domain of the SARS-CoV-2 S1-Subunit of the Spike Protein: A Population-Based Study. *Journal of Clinical Medicine*. 2020;9(12):3989. doi:[10.3390/jcm9123989](https://doi.org/10.3390/jcm9123989).

102. Storgaard SF, Eiset AH, Abdullahi F, Wejse C. [First wave of COVID-19 did not reach the homeless population in Aarhus](https://www.ncbi.nlm.nih.gov/pubmed/33269697). *Danish Medical Journal*. 2020;67(12).

103. Thomas SN, Altawallbeh G, Zaun CP, et al. Initial determination of COVID-19 seroprevalence among outpatients and healthcare workers in Minnesota using a novel SARS-CoV-2 total antibody ELISA. *Clinical Biochemistry*. February 2021. doi:[10.1016/j.clinbiochem.2021.01.010](https://doi.org/10.1016/j.clinbiochem.2021.01.010).

104. Kahlert CR, Persi R, Güsewell S, et al. Non-occupational and occupational factors associated with specific SARS-CoV-2 antibodies among hospital workers – A multicentre cross-sectional study. *Clinical Microbiology and Infection*. 2021;0(0). doi:[10.1016/j.cmi.2021.05.014](https://doi.org/10.1016/j.cmi.2021.05.014).

105. Griffin J, Tully E, Cody F, et al. Persistence of SARS-CoV-2 antibodies and symptoms in an Irish Healthcare Worker (HCW) setting: Results of the COVID Antibody Staff Testing (CAST) Study. *medRxiv*. February 2021:2021.02.10.20248323. doi:[10.1101/2021.02.10.20248323](https://doi.org/10.1101/2021.02.10.20248323).

106. Klevebro S, Bahram F, Elfström KM, et al. Risk of SARS-CoV-2 exposure among hospital healthcare workers in relation to patient contact and type of care. *medRxiv*. January 2021:2021.01.28.21250664. doi:[10.1101/2021.01.28.21250664](https://doi.org/10.1101/2021.01.28.21250664).

107. Poustchi H, Darvishian M, Mohammadi Z, et al. SARS-CoV-2 antibody seroprevalence in the general population and high-risk occupational groups across 18 cities in Iran: A population-based cross-sectional study. *The Lancet Infectious Diseases*. 2021;21(4):473-481. doi:[10.1016/S1473-3099(20)30858-6](https://doi.org/10.1016/S1473-3099(20)30858-6).

108. Brunner WM, Hirabayashi L, Krupa NL, et al. Severe acute respiratory coronavirus virus 2 (SARS-CoV-2) IgG results among healthcare workers in a rural upstate New York hospital system. *Infection Control & Hospital Epidemiology*. October 2020:1-4. doi:[10.1017/ice.2020.1296](https://doi.org/10.1017/ice.2020.1296).

109. Cleto-Yamane TL, Rodrigues-Santos G, Magalhães-Barbosa MC de, et al. Screening of COVID-19 in outpatient children with cancer or solid organ transplantation: Preliminary report. *European Journal of Pediatrics*. March 2021. doi:[10.1007/s00431-021-04044-9](https://doi.org/10.1007/s00431-021-04044-9).

110. Ariza B, Torres X, Salgado D, et al. Seroprevalence and seroconversion rates to SARS-CoV-2 in interns, residents, and medical doctors in a University Hospital in Bogotá, Colombia. *Infectio*. 2021;25(3):145-152. doi:[10.22354/in.v25i3.938](https://doi.org/10.22354/in.v25i3.938).

111. Gonzalez JM, Santos-Barbosa JC, Jaller C, et al. Seroreactivity to SARS-CoV-2 in individuals attending a university campus in Bogotá Colombia. *medRxiv*. March 2021:2021.03.15.21253609. doi:[10.1101/2021.03.15.21253609](https://doi.org/10.1101/2021.03.15.21253609).

112. Chang L, Hou W, Zhao L, et al. The prevalence of antibodies to SARS-CoV-2 among blood donors in China. *medRxiv*. July 2020:32. doi:[10.1101/2020.07.13.20153106](https://doi.org/10.1101/2020.07.13.20153106).

113. City of Holyoke government. *City of Holyoke Serosurvey Report*. Holyoke, MA; 2021. <https://www.holyoke.org/documents/city-of-holyoke-serosurvey-report/>.

114. Region TS. *13 April*. The Stockholm Region; 2021. <https://www.regionstockholm.se/verksamhet/halsa-och-vard/nyheter-lagesrapporter-covid-19/2021/04/13-april-lagesrapport-om-covid-19/>. Accessed November 23, 2021.

115. Fenton F, Stokes S, Eagleton M. A cross-section observational study on the seroprevalence of antibodies to COVID-19 in patients receiving opiate agonist treatment. *Irish Journal of Medical Science (1971 -)*. July 2021. doi:[10.1007/s11845-021-02660-w](https://doi.org/10.1007/s11845-021-02660-w).

116. Schubert L, Strassl R, Burgmann H, et al. A Longitudinal Seroprevalence Study Evaluating Infection Control and Prevention Strategies at a Large Tertiary Care Center with Low COVID-19 Incidence. *International Journal of Environmental Research and Public Health*. 2021;18(8):4201. doi:[10.3390/ijerph18084201](https://doi.org/10.3390/ijerph18084201).

117. Martino RJ, Krause KD, Griffin M, et al. A Nationwide Survey of COVID-19 Testing in LGBTQ+ Populations in the United States. *Public Health Reports*. 2021;136(4):493-507. doi:[10.1177/00333549211018190](https://doi.org/10.1177/00333549211018190).

118. Sughayer MA, Mansour A, Nuirat AA, Souan L, Abdel-Razeq R, Siag M. A second dramatic rise in seroprevalence rates of SARS-CoV-2 antibodies among adult healthy blood donors in Jordan; have we achieved herd immunity? *medRxiv*. August 2021. doi:[10.1101/2021.08.15.21261584](https://doi.org/10.1101/2021.08.15.21261584).

119. Bellizzi S, Alsawalha L, Sheikh Ali S, et al. A three-phase population based sero-epidemiological study: Assessing the trend in prevalence of SARS-CoV-2 during COVID-19 pandemic in Jordan. *One Health*. 2021;13:100292. doi:<https://doi.org/10.1016/j.onehlt.2021.100292>.

120. Holt SG, Mahmoud S, Ahmed W, et al. An analysis of antibody responses and clinical sequalae of the Sinopharm HB02 COVID19 vaccine in dialysis patients in the United Arab Emirates. *Nephrology*. 2021;n/a(n/a). doi:[10.1111/nep.13980](https://doi.org/10.1111/nep.13980).

121. Zhou K, Blanc-Lapierre A, Seegers V, et al. Anosmia but Not Ageusia as a COVID-19-Related Symptom among Cancer Patients—First Results from the PAPESCO-19 Cohort Study. *Cancers*. 2021;13(14):3389. doi:[10.3390/cancers13143389](https://doi.org/10.3390/cancers13143389).

122. Rubbert-Roth A, Vuilleumier N, Ludewig B, et al. Anti-S1 antibodies after vaccination with anti SARS-CoV-2 mRNA vaccines in patients with rheumatoid arthritis differ in magnitude and kinetics from healthy controls: Results from a prospective, observational controlled study. In: *Abstracts of the Annual Congress of the Swiss Society of Rheumatology*. Vol 151. Lausanne, Switzerland: Swiss Medical Weekly; 2021. doi:[10.4414/SMW.2021.w30056](https://doi.org/10.4414/SMW.2021.w30056).

123. Elli L, Facciotti F, Lombardo V, et al. Anti-SARS-CoV-2 immunoglobulin profile in patients with celiac disease living in a high incidence area. *Digestive and Liver Disease*. September 2021:S1590865821007696. doi:[10.1016/j.dld.2021.08.027](https://doi.org/10.1016/j.dld.2021.08.027).

124. Zejda JE, Kowalska M, Brożek GM, Barański K, Kaleta-Pilarska A. Infection Fatality Rate (IFR) during the course of COVID-19 pandemic in Upper Silesia Metropolitan Area (Poland) in 2020. *Medycyna Pracy*. November 2021. doi:[10.13075/mp.5893.01179](https://doi.org/10.13075/mp.5893.01179).

125. Eichner FA, Gelbrich G, Weißbrich B, et al. Seroprävalenz von COVID-19 und psychosoziale Auswirkungen in der Allgemeinbevölkerung: Ergebnisse des STAAB-COVID-One Programms. *Das Gesundheitswesen*. 2021;83(12):965-975. doi:[10.1055/a-1630-7601](https://doi.org/10.1055/a-1630-7601).

126. Levorson RE, Christian E, Hunter B, et al. A cross-sectional investigation of SARS-CoV-2 seroprevalence and associated risk factors in children and adolescents in the United States. *PLOS ONE*. 2021;16(11):e0259823. doi:[10.1371/journal.pone.0259823](https://doi.org/10.1371/journal.pone.0259823).

127. Di Ruscio M, Lunardi G, Buonfrate D, et al. A Seroprevalence Study of Anti-SARS-CoV-2 Antibodies in Patients with Inflammatory Bowel Disease during the Second Wave of the COVID-19 Pandemic in Italy. *Medicina*. 2021;57(10):1048. doi:[10.3390/medicina57101048](https://doi.org/10.3390/medicina57101048).

128. Syed MA, A/Qotba HA, Al Nuaimi AS, et al. Antibody Response to SARS-CoV-2: A Cohort Study in Qatar’s Primary Care Settings. *Journal of Primary Care & Community Health*. 2021;12:215013272110505. doi:[10.1177/21501327211050569](https://doi.org/10.1177/21501327211050569).

129. Matta J, Wiernik E, Robineau O, et al. Association of Self-reported COVID-19 Infection and SARS-CoV-2 Serology Test Results With Persistent Physical Symptoms Among French Adults During the COVID-19 Pandemic. *JAMA Internal Medicine*. 2022;182(1):19-25. doi:[10.1001/jamainternmed.2021.6454](https://doi.org/10.1001/jamainternmed.2021.6454).

130. Hori H, Fukuchi T, Sanui M, Moriya T, Sugawara H. Comprehensive infection control measures prevent hospital-acquired severe acute respiratory syndrome coronavirus 2 infection: A single-center prospective cohort study and seroprevalence survey. *PLOS ONE*. 2021;16(10):e0257513. doi:[10.1371/journal.pone.0257513](https://doi.org/10.1371/journal.pone.0257513).

131. Weinbrand-Goichberg J, Ben Shalom E, Rinat C, et al. COVID-19 in children and young adults with kidney disease: Risk factors, clinical features and serological response. *Journal of Nephrology*. October 2021. doi:[10.1007/s40620-021-01171-2](https://doi.org/10.1007/s40620-021-01171-2).

132. Islamoglu MS, Cengiz M, Borku Uysal B, et al. COVID-19 seroconversion in the aircrew from Turkey. *Travel Medicine and Infectious Disease*. 2021;44:102190. doi:[10.1016/j.tmaid.2021.102190](https://doi.org/10.1016/j.tmaid.2021.102190).

133. Canadian Blood Services. *COVID-19 Seroprevalence Report October 13, 2021 Report #13 - August 2021 Survey Tracking Seroprevalence in the Vaccine Era*.; 2021.

134. Talaei M, Faustini S, Holt H, et al. *Determinants of Pre-Vaccination Antibody Responses to SARS-CoV-2*. Rochester, NY: Social Science Research Network; 2021. doi:[10.2139/ssrn.3944582](https://doi.org/10.2139/ssrn.3944582).

135. Brehm TT, Ullrich F, Thompson M, et al. *Three Separate Spike Antigen Exposures by COVID-19 Vaccination or SARS-CoV-2 Infection Elicit Strong Humoral Immune Responses in Healthcare Workers*. medRxiv; 2022:2022.03.06.22271718. doi:[10.1101/2022.03.06.22271718](https://doi.org/10.1101/2022.03.06.22271718).

136. Kleynhans J, Tempia S, Wolter N, et al. SARS-CoV-2 Seroprevalence after Third Wave of Infections, South Africa. *Emerging Infectious Diseases*. 2022;28(5). doi:[10.3201/eid2805.220278](https://doi.org/10.3201/eid2805.220278).

137. Goto A, Miyakawa K, Nakayama I, et al. *Analysis of Humoral Immunity Against Emerging SARS-CoV-2 Variants*. medRxiv; 2022:2022.03.26.22272766. doi:[10.1101/2022.03.26.22272766](https://doi.org/10.1101/2022.03.26.22272766).

138. Graciaa DS, Kempker RR, Wang YF (Wayne), et al. SARS-CoV-2 seroprevalence among healthcare personnel at a large health system in Atlanta. *The American Journal of the Medical Sciences*. April 2022:S0002962922001586. doi:[10.1016/j.amjms.2022.04.009](https://doi.org/10.1016/j.amjms.2022.04.009).

139. Canadian Blood Services. *COVID-19 Seroprevalence Report April 10th, 2022 Report #19*.; 2022. [N/A](https://n/A).

140. Ahava MJ, Jarva H, Jääskeläinen AJ, Lappalainen M, Vapalahti O, Kurkela S. Rapid increase in SARS-CoV-2 seroprevalence during the emergence of Omicron variant, Finland. *European Journal of Clinical Microbiology & Infectious Diseases*. 2022;41(6):997-999. doi:[10.1007/s10096-022-04448-x](https://doi.org/10.1007/s10096-022-04448-x).

141. Suemori K, Taniguchi Y, Okamoto A, et al. Two-year seroprevalence surveys of SARS-CoV-2 antibodies among outpatients and healthcare workers in Japan. *Japanese Journal of Infectious Diseases*. 2022;75(5):523-526. doi:[10.7883/yoken.JJID.2022.155](https://doi.org/10.7883/yoken.JJID.2022.155).

142. Leon‐Sicairos N, Angulo‐Zamudio UA, Pacheco‐Avila M, et al. Epidemiological and clinical characteristics of pregnant women and neonates with COVID‐19 in Northwest Mexico. *American Journal of Reproductive Immunology*. June 2022. doi:[10.1111/aji.13583](https://doi.org/10.1111/aji.13583).

143. Sugiyama A, Okada F, Abe K, et al. A longitudinal study of anti-SARS-CoV-2 antibody seroprevalence in a random sample of the general population in Hiroshima in 2020. *Environmental Health and Preventive Medicine*. 2022;27(0):30-30. doi:[10.1265/ehpm.22-00016](https://doi.org/10.1265/ehpm.22-00016).

144. Public Health Scotland. Enhanced Surveillance of COVID-19 in Scotland - Population-based seroprevalence surveillance 20 July 2022. *Public Health Scotland*. July 2022. doi:[10.52487/89815](https://doi.org/10.52487/89815).

145. Olea A, Matute I, Hirmas M, et al. Immune response against SARS-CoV-2 of primary healthcare personnel in a commune of Santiago, Chile: Follow-up at 6 months. *medRxiv*. August 2022. doi:[10.1101/2022.08.03.22278369](https://doi.org/10.1101/2022.08.03.22278369).

146. Awandu SS, Ochieng A, Onyango B, et al. High seroprevalence of Immunoglobulin G (IgG) and IgM antibodies to SARS-CoV-2 in asymptomatic and symptomatic individuals amidst vaccination roll-out in western Kenya. July 2022. doi:[10.1101/2022.07.27.22278095](https://doi.org/10.1101/2022.07.27.22278095).

147. Saul Eric Mwale, Master Chisale, McLenelious Chimenya, et al. Prevalence of Immunoglobulin (G (IgG) and M (IgM)) Against SARS-COV-2 and risk factors for positivity among Students at tertiary education institution, Malawi. August 2022. doi:[10.21203/rs.3.rs-1870665/v1](https://doi.org/10.21203/rs.3.rs-1870665/v1).

148. Denina M, Trada M, Tinti D, et al. Increase in newly diagnosed type 1 diabetes and serological evidence of recent SARS-CoV-2 infection: Is there a connection? *Frontiers in Medicine*. 2022;9. <https://www.frontiersin.org/articles/10.3389/fmed.2022.927099>. Accessed August 24, 2022.

149. Shukri AMA, Adnan A, Mui WS, et al. Seroprevalence of SARS-CoV-2 Antibodies Among Blood Donors in Malaysia During the Pre-Vaccination Period. *International Journal of Infectious Diseases*. 2022;116:S33-S34. doi:[10.1016/j.ijid.2021.12.080](https://doi.org/10.1016/j.ijid.2021.12.080).

150. Lee CC, Segaloff HE, Cole D, et al. A cohort study measuring SARS-CoV-2 seroconversion and serial viral testing in university students. *BMC Infectious Diseases*. 2022;22(1):314. doi:[10.1186/s12879-022-07314-5](https://doi.org/10.1186/s12879-022-07314-5).

151. Anand SS, Arnold C, Bangdiwala S, et al. *What Factors Converged to Create a COVID-19 Hot-Spot?* medRxiv; 2022:2022.04.01.22273252. doi:[10.1101/2022.04.01.22273252](https://doi.org/10.1101/2022.04.01.22273252).

152. Clarke KEN, Kim Y, Jones J, et al. Pediatric Infection-Induced SARS-CoV-2 Seroprevalence Estimation Using Commercial Laboratory Specimens: How Representative Is It of the General U.S. Pediatric Population? *SSRN*. May 2022. doi:[10.2139/ssrn.4092074](https://doi.org/10.2139/ssrn.4092074).

153. Bahlawan O, Badra R, Semaan H, et al. Prevalence and determinants of SARS-CoV-2 neutralizing antibodies in Lebanon. *Archives of Virology*. 2022;167(7):1509-1519. doi:[10.1007/s00705-022-05470-2](https://doi.org/10.1007/s00705-022-05470-2).

154. Nash D, Rane MS, Robertson MM, et al. SARS-CoV-2 incidence and risk factors in a national, community-based prospective cohort of U.S. adults. *Clinical Infectious Diseases*. May 2022:ciac423. doi:[10.1093/cid/ciac423](https://doi.org/10.1093/cid/ciac423).

155. Al-Saray D, Al-Asady FM, Tuhair T. Role of Antibodies against SARS-COV-2 in the Detection of Corona Virus, its Transmissibility and Immunological Status Determination among Different Population in Babylon Province. *Open Access Macedonian Journal of Medical Sciences*. 2022;10(A):644-649. doi:[10.3889/oamjms.2022.9372](https://doi.org/10.3889/oamjms.2022.9372).

156. Salum SS, Sheikh MA, Hebestreit A, Kelm S. Anti SARS-CoV2 seroprevalence in Zanzibar in 2021 before the Omicron wave. *IJID Regions*. July 2022. doi:[10.1016/j.ijregi.2022.06.007](https://doi.org/10.1016/j.ijregi.2022.06.007).

157. Ferreira MU, Giacomini I, Sato PM, et al. SARS-CoV-2 seropositivity and COVID-19 among 5 years-old Amazonian children and their association with poverty and food insecurity. Pappas G, ed. *PLOS Neglected Tropical Diseases*. 2022;16(7):e0010580. doi:[10.1371/journal.pntd.0010580](https://doi.org/10.1371/journal.pntd.0010580).

158. Coelho LE, Luz PM, Pires DC, et al. Prevalence and predictors of anti-SARS-CoV-2 serology in a highly vulnerable population of Rio de Janeiro: A population-based serosurvey. *The Lancet Regional Health - Americas*. 2022;15:100338. doi:[10.1016/j.lana.2022.100338](https://doi.org/10.1016/j.lana.2022.100338).

159. Terças-Trettel ACP, Muraro AP, Andrade AC de S, Oliveira EC de. Self-reported symptoms and seroprevalence against SARS-CoV-2 in the population of Mato Grosso: A household-based survey in 2020. *Revista da Associação Médica Brasileira*. 2022;68:928-934. doi:[10.1590/1806-9282.20220078](https://doi.org/10.1590/1806-9282.20220078).

160. Abdelmoniem R, Fouad R, Shawky S, et al. SARS-CoV-2 infection among asymptomatic healthcare workers of the emergency department in a tertiary care facility. *Journal of Clinical Virology*. 2021;134:104710. doi:[10.1016/j.jcv.2020.104710](https://doi.org/10.1016/j.jcv.2020.104710).

161. Lucinde R, Mugo D, Bottomley C, et al. *Sero-Surveillance for IgG to SARS-CoV-2 at Antenatal Care Clinics in Three Kenyan Referral Hospitals*. medRxiv; 2022:2022.03.03.22271860. doi:[10.1101/2022.03.03.22271860](https://doi.org/10.1101/2022.03.03.22271860).

162. Wolter N, Tempia S, Gottberg A von, et al. Seroprevalence of SARS-CoV-2 after the second wave in South Africa in HIV-infected and uninfected persons: A cross-sectional household survey. *Clinical Infectious Diseases*. March 2022. doi:[10.1093/cid/ciac198](https://doi.org/10.1093/cid/ciac198).

163. Fernández-Rojas MA, Luna-Ruiz Esparza MA, Campos-Romero A, et al. Seroconversion dynamic and SARS-CoV-2 seropositivity in unvaccinated population during the first and second outbreaks in Mexico. *Scientific Reports*. 2022;12(1):5241. doi:[10.1038/s41598-022-09395-3](https://doi.org/10.1038/s41598-022-09395-3).

164. Alenazi MW, Algaisi A, Zowawi HM, Aldibasi O, Hashem AM, Khalaf Alharbi N. Seroprevalence of COVID-19 in Riyadh City during the early increase of COVID-19 infections in Saudi Arabia, June 2020. *Saudi Journal of Biological Sciences*. April 2022:103282. doi:[10.1016/j.sjbs.2022.103282](https://doi.org/10.1016/j.sjbs.2022.103282).

165. Kim H, Schultz-Heienbrok R, Uhle M, et al. Longitudinal study of SARS-CoV-2 infections in different employee groups of long distance train services from June 2020 until February 2021 in Germany. *Epidemiology and Infection*. 2022;150:e88. doi:[10.1017/S095026882200070X](https://doi.org/10.1017/S095026882200070X).

166. Bueno-Hernández N, Carrillo-Ruíz JD, Méndez-García LA, et al. High Incidence Rate of SARS-CoV-2 Infection in Health Care Workers at a Dedicated COVID-19 Hospital: Experiences of the Pandemic from a Large Mexican Hospital. *Healthcare*. 2022;10(5):896. doi:[10.3390/healthcare10050896](https://doi.org/10.3390/healthcare10050896).

167. Hijazi MHA, Jeffree MS, Pang NTP, et al. Seroprevalence of COVID-19 and Psychological Distress among Front Liners at the Universiti Malaysia Sabah Campus during the Third Wave of COVID-19. *International Journal of Environmental Research and Public Health*. 2022;19(11):6840. doi:[10.3390/ijerph19116840](https://doi.org/10.3390/ijerph19116840).

168. Morrone A, Buonomini AR, Sannella A, Pimpinelli F, Rotulo A. Unequal Access to Testing and Vaccination Services for the Homeless and Undocumented Population During COVID-19 Pandemic. *International Journal of Public Health*. 2022;67. doi:[10.3389/ijph.2022.1604851](https://doi.org/10.3389/ijph.2022.1604851).

169. Abdullahi A, Oladele D, Owusu M, et al. SARS-CoV-2 Antibody Responses to AZD1222 Vaccination in West Africa. *Research Square*. July 2022. doi:[10.21203/rs.3.rs-1834968/v1](https://doi.org/10.21203/rs.3.rs-1834968/v1).

170. Coyer L, Boyd A, Schinkel J, et al. SARS-CoV-2 antibody prevalence and correlates of six ethnic groups living in Amsterdam, the Netherlands: A population-based cross-sectional study, June–October 2020. *BMJ Open*. 2022;12(1):e052752. doi:[10.1136/bmjopen-2021-052752](https://doi.org/10.1136/bmjopen-2021-052752).

171. Forster J, Streng A, Rudolph P, et al. Feasibility of SARS-CoV-2 Surveillance Testing Among Children and Childcare Workers at German Day Care Centers: A Nonrandomized Controlled Trial. *JAMA Network Open*. 2022;5(1):e2142057. doi:[10.1001/jamanetworkopen.2021.42057](https://doi.org/10.1001/jamanetworkopen.2021.42057).

172. Mori K, Imaki S, Ohyama Y, Satoh K, Abe T, Takeuchi I. Rapid screening for severe acute respiratory syndrome coronavirus 2 infection with a combined point-of-care antigen test and an immunoglobulin G antibody test. *PLOS ONE*. 2022;17(2):e0263327. doi:[10.1371/journal.pone.0263327](https://doi.org/10.1371/journal.pone.0263327).

173. Zhang J, Liu J, Li N, et al. Serological detection of 2019-nCoV respond to the epidemic: A useful complement to nucleic acid testing. *medRxiv*. March 2020:2020.03.04.20030916. doi:[10.1101/2020.03.04.20030916](https://doi.org/10.1101/2020.03.04.20030916).

174. Heireman L, Baetens M, Menten B, Dehaene I, Padalko E. Comparison of the positivity rate of anti-spike and anti-nucleocapsid SARS-CoV-2 IgG in asymptomatic pregnant women. *Journal of Obstetrics and Gynaecology*. December 2021:1-2. doi:[10.1080/01443615.2021.1997960](https://doi.org/10.1080/01443615.2021.1997960).

175. Zar HJ, MacGinty R, Workman L, et al. Natural and hybrid immunity following four COVID-19 waves: A prospective cohort study of mothers in South Africa. *eClinicalMedicine*. 2022;53:101655. doi:[10.1016/j.eclinm.2022.101655](https://doi.org/10.1016/j.eclinm.2022.101655).

176. Guseva Canu I, Hemmendinger M, Toto A, et al. Oxidative Potential in Exhaled Air (OPEA) as a Tool for Predicting Certain Respiratory Disorders in the General Adult Population: Cross-Sectional Analysis Nested in the Swiss Health Study. *Antioxidants*. 2022;11(10):2079. doi:[10.3390/antiox11102079](https://doi.org/10.3390/antiox11102079).

177. Anzinger JJ, Cameron-McDermott SM, Phillips YZR, et al. Prevalence of SARS-CoV-2 Antibodies after the Omicron Surge, Kingston, Jamaica, 2022. *medRxiv*. September 2022:2022.09.20.22280173. doi:[10.1101/2022.09.20.22280173](https://doi.org/10.1101/2022.09.20.22280173).

178. Rahman M, Khan SR, Alamgir ASM, et al. Seroprevalence of SARS-CoV-2 antibodies among Forcibly Displaced Myanmar Nationals in Cox’s Bazar, Bangladesh 2020: A population-based cross-sectional study. *BMJ Open*. 2022;12(11):e066653. doi:[10.1136/bmjopen-2022-066653](https://doi.org/10.1136/bmjopen-2022-066653).

179. Graciaa DS, Verkerke HP, Guarner J, et al. Estimating severe acute respiratory coronavirus virus 2 (SARS-CoV-2) seroprevalence from residual clinical blood samples, January–March 2021. *Antimicrobial Stewardship & Healthcare Epidemiology*. 2022;2(1):e159. doi:[10.1017/ash.2022.298](https://doi.org/10.1017/ash.2022.298).

180. Kirby Institute. Seroprevalence of SARS-CoV-2-specific antibodies among Australian blood donors: Round 3 update. *Kirby Institute*. November 2022. <https://kirby.unsw.edu.au/sites/default/files/COVID19-Blood-Donor-Report-Round3-Aug-Sep-2022.pdf>. Accessed November 14, 2022.

181. Yang M-H, Lai J, Chen Y-Y, et al. Surveillance of Sars-Cov-2 Virus and Its Corresponding Antibody in Blood Donors after a Nosocomial Infection in Taiwan. In: *2022 AABB Annual Meeting Plenary, Oral, and Poster Abstract Presentations*. Vol 62. Orlando, Florida: Transfusion; 2022:203A-204A. doi:[10.1111/trf.17058](https://doi.org/10.1111/trf.17058).

182. Aubry M, Maset N, Chapman L, et al. Seroprevalence of SARS-CoV-2 Antibodies in French Polynesia and Perspective for Vaccine Strategies. *Preprints*. December 2022. doi:[10.20944/preprints202212.0386.v1](https://doi.org/10.20944/preprints202212.0386.v1).

183. Bassal R, Keinan-Boker L, Cohen D, Mendelson E, Lustig Y, Indenbaum V. Estimated Infection and Vaccine Induced SARS-CoV-2 Seroprevalence in Israel among Adults, January 2020–July 2021. *Vaccines*. 2022;10(10):1663. doi:[10.3390/vaccines10101663](https://doi.org/10.3390/vaccines10101663).

184. Akinbami LJ, Kruszon-Moran D, Wang C-Y, et al. SARS-CoV-2 Serology and Self-Reported Infection Among Adults - National Health and Nutrition Examination Survey, United States, August 2021-May 2022. *MMWR Morbidity and Mortality Weekly Report*. 2022;71(48):1522-1525. doi:[10.15585/mmwr.mm7148a4](https://doi.org/10.15585/mmwr.mm7148a4).

185. Hopper L. Early antibody testing suggests COVID-19 infections in L.A. County greatly exceed documented cases. *USC News*. April 2020. <https://news.usc.edu/168987/antibody-testing-results-covid-19-infections-los-angeles-county/>. Accessed March 13, 2021.

186. Cherenack EM, Salazar AS, Nogueira NF, et al. Infection with SARS-CoV-2 is associated with menstrual irregularities among women of reproductive age. Njenga MK, ed. *PLOS ONE*. 2022;17(10):e0276131. doi:[10.1371/journal.pone.0276131](https://doi.org/10.1371/journal.pone.0276131).

187. Ginman B, Pahnke S, Freyhult E, et al. 1427P The role of anxiety and self-isolation in seropositivity for COVID-19 in actively treated cancer patients in Sweden. In: *Abstract Book of the ESMO Congress 2022*. Vol 33. Annals of oncology; 2022:S1197-S1198. doi:[10.1016/j.annonc.2022.07.1530](https://doi.org/10.1016/j.annonc.2022.07.1530).

188. Frei A, Kaufmann M, Amati R, et al. Development of hybrid immunity during a period of high incidence of infections with Omicron subvariants: A prospective population based multi-region cohort study. *medRxiv*. October 2022. doi:[10.1101/2022.10.14.22281076](https://doi.org/10.1101/2022.10.14.22281076).

189. Sheikh Ali S, Kheirallah KA, Sharkas G, et al. SARS-CoV-2 Seroepidemiological Investigation in Jordan: Seroprevalence, Herd Immunity, and Vaccination Coverage. A Population-Based National Study. *International Journal of General Medicine*. 2022;15:7053-7062. doi:[10.2147/IJGM.S371711](https://doi.org/10.2147/IJGM.S371711).

190. Pisani LF, Mola S, Crespi G, et al. Serum proteomics and metabolomics study on susceptibility to SARS-CoV-2 infection in IBD patients. In: *UEG Week 2022 Moderated Posters*. Vol 10. United European Gastroenterology Journal; 2022:240. doi:[10.1002/ueg2.12294](https://doi.org/10.1002/ueg2.12294).

191. Bhuiyan TR, Akhtar M, Akter A, et al. Seroprevalence of SARS-CoV-2 antibodies in Bangladesh related to novel coronavirus infection. *IJID Regions*. 2022;2:198-203. doi:[10.1016/j.ijregi.2022.01.013](https://doi.org/10.1016/j.ijregi.2022.01.013).

192. Moyano LM, Toledo AK, Chirinos J, et al. SARS-CoV-2 seroprevalence on the north coast of Peru: A cross-sectional study after the first wave. *medRxiv*. September 2022. doi:[10.1101/2022.09.07.22279669](https://doi.org/10.1101/2022.09.07.22279669).

193. Nasimiyu C, Ngere I, Dawa J, et al. Near-Complete SARS-CoV-2 Seroprevalence among Rural and Urban Kenyans despite Significant Vaccine Hesitancy and Refusal. *Vaccines*. 2023;11(1):68. doi:[10.3390/vaccines11010068](https://doi.org/10.3390/vaccines11010068).

194. Dumont R, Richard V, Lorthe E, et al. A population-based serological study of post-COVID syndrome prevalence and risk factors in children and adolescents. *Nature Communications*. 2022;13(1):7086. doi:[10.1038/s41467-022-34616-8](https://doi.org/10.1038/s41467-022-34616-8).

195. Wagatsuma K, Yoshioka S, Yamazaki S, et al. Assessing the Pre-Vaccination Anti-SARS-CoV-2 IgG Seroprevalence among Residents and Staff in Nursing Home in Niigata, Japan, November 2020. *Viruses*. 2022;14(11):2581. doi:[10.3390/v14112581](https://doi.org/10.3390/v14112581).

196. Gigot C, Pisanic N, Kruczynski K, et al. SARS-CoV-2 antibody prevalence among industrial livestock operation workers and nearby community residents, North Carolina, USA, 2021-2022. *medRxiv*. November 2022. doi:[10.1101/2022.10.31.22281764](https://doi.org/10.1101/2022.10.31.22281764).

197. Evaristo M, Santos E, Borges J, Covas D, Kashima S. O impacto do SARS-CoV-2 na medicina transfusional: Avaliação da soroprevalência e detecção molecular de SARS-CoV-2 em doadores de sangue. *Hematology, Transfusion and Cell Therapy*. 2022;44:S663-S664. doi:[10.1016/j.htct.2022.09.1139](https://doi.org/10.1016/j.htct.2022.09.1139).

198. Rodriguez A. MGH Coronavirus Case Study In Chelsea Will Help Researchers Understand Immunity. *CBS Boston*. April 2020. <https://boston.cbslocal.com/2020/04/23/coronavirus-mgh-case-study-john-iafrate-herd-immunity/>. Accessed March 13, 2021.

199. Health of Israel M of. *COVID-19 הסיקור הסרו-אפידמיולוגי הלאומי ל*. Ministry of Health of Israel; 2020. <https://www.gov.il/BlobFolder/reports/de-covid19-28062020-17092020/he/files_publications_corona_DE-covid19.pdf>. Accessed May 14, 2021.
